# NOTCH1 is critical for fibroblast-mediated induction of cardiomyocyte specialization into ventricular conduction system-like cells in vitro

**DOI:** 10.1038/s41598-020-73159-0

**Published:** 2020-09-30

**Authors:** Agatha Ribeiro da Silva, Elida A. Neri, Lauro Thiago Turaça, Rafael Dariolli, Miriam H. Fonseca-Alaniz, Artur Santos-Miranda, Danilo Roman-Campos, Gabriela Venturini, Jose E. Krieger

**Affiliations:** 1 Lab Genetics & Molec Cardiology, Instituto do Coracao (InCor) da Faculdade de Medicina da Universidade de Sao Paulo (FMUSP), São Paulo, Brazil; 2 Paulista School of Medicine, Federal University of São Paulo (EPM-UNIFESP), São Paulo, Brazil

**Keywords:** Cardiovascular biology, Cell biology

## Abstract

Cardiac fibroblasts are present throughout the myocardium and are enriched in the microenvironment surrounding the ventricular conduction system (VCS). Several forms of arrhythmias are linked to VCS abnormalities, but it is still unclear whether VCS malformations are cardiomyocyte autonomous or could be linked to crosstalk between different cell types. We reasoned that fibroblasts influence cardiomyocyte specialization in VCS cells. We developed 2D and 3D culture models of neonatal rat cardiac cells to assess the influence of cardiac fibroblasts on cardiomyocytes. Cardiomyocytes adjacent to cardiac fibroblasts showed a two-fold increase in expression of VCS markers (NAV1.5 and CONTACTIN 2) and calcium transient duration, displaying a Purkinje-like profile. Fibroblast-conditioned media (fCM) was sufficient to activate VCS-related genes (*Irx3*, *Scn5a*, Connexin 40) and to induce action potential prolongation, a hallmark of Purkinge phenotype. fCM-mediated response seemed to be spatially-dependent as cardiomyocyte organoids treated with fCM had increased expression of connexin 40 and NAV1.5 primarily on its outer surface. Finally, NOTCH1 activation in both cardiomyocytes and fibroblasts was required for connexin 40 up-regulation (a proxy of VCS phenotype). Altogether, we provide evidence that cardiac fibroblasts influence cardiomyocyte specialization into VCS-like cells via NOTCH1 signaling in *vitro.*

## Introduction

Fibroblasts are one of the most abundant non-myocyte cells in the heart^1^. They are primarily known for their role in extracellular matrix homeostasis and remodeling during disease progression, but recent evidence suggests their influence in cardiomyocyte embryonic development and adult hypertrophy, as well as stabilization of cardiomyocyte contraction through heterocellular coupling^2,3^.

Cardiac fibroblasts are present throughout the myocardium and are enriched in the microenvironment surrounding the ventricular conduction system (VCS)^4–6^. The VCS is a specialized structure responsible for conduction of electrical potentials throughout the ventricles^7,8^. The VCS initiates in the His bundle and extends to the peripheral ventricular conduction system (PVCS) composed of rapidly conducting fibers, known as Purkinje fibers^9^. In the embryonic heart, the VCS originates from myocytes in the trabecular myocardium, which also give rise to the adjacent working myocardium, and its postnatal maturation is crucial for proper cardiac function^10–12^.

Several forms of acquired and inherited arrhythmias have been associated with abnormalities in the VCS, which are related to sudden cardiac death^13–15^. Recent studies have contributed to the understanding of VCS development through lineage tracing analyses, the VCS gene network, and signaling pathways^7,8,16,17^. The VCS gene network is primarily regulated by the cardiogenic transcription factors *Irx3* and *Nkx2.5*^10,12,18–21^ . These transcription factors physically interact and are required for myocyte recruitment to differentiate into fast conducting fibers and PVCS postnatal maturation^10,12,19,22,23^.

Activation of NOTCH1 pathway has also been shown to induce specialization of murine cardiomyocytes into the conduction phenotype in vivo and in vitro*,* during embryonic development and postnatal stages^24^ . The importance of NOTCH1 signaling to the development of the conduction system appears to be conserved among species, since morpholino knockdown of Notch1b in zebrafish results in defective atrioventricular conduction^25^. Recently, the NOTCH pathway has been pointed as an integrating pathway, being responsive to a variety of factors and events such as shear stress, hypoxia and especially the composition of the extracellular matrix, which is directly influenced by cardiac fibroblasts^26^. In fact, cardiac fibroblasts secrete various extracellular matrix components that have been shown to influence cardiomyocytes during embryonic development^2,27,28^. Based on the current state of knowledge, whether cardiomyocyte specialization into VCS cells is a cell autonomous event or requires cellular crosstalk with other cell types is yet to be determined.

Considering that cardiac fibroblasts are enriched in the adjacent areas of the ventricular conduction system, we hypothesized that they play a role in the differentiation of cardiomyocytes into rapidly conducting fibers that compose the VCS by juxtacrine signaling (e.g., NOTCH pathway, interactions with the extracellular matrix or gap junction-mediated signaling). To test this hypothesis, we evaluated the molecular and functional properties of neonatal rat cardiomyocytes co-cultured with cardiac fibroblasts or treated with cardiac fibroblast-conditioned media in 2D and 3D culture models. Our findings provide evidence that cardiac fibroblast-secreted factors induce the expression of the VCS markers as well as functional changes in cardiomyocytes towards a VCS-like phenotype in vitro. This response was dependent on the activation of NOTCH1 pathway in both cardiomyocytes and fibroblasts.

## Methods

### Animals

All animal experiments were conducted in accordance with guidelines from the Directive 2010/63/EU of the European Parliament on animal experimentation in compliance with European guidelines and International Laws and Policies (EC Council Directive 86/609, OJL 34, 12 December 1987) and were approved by the Ethics Committee in Animal Research (CEUA) of the University of Sao Paulo (097/16).

### Isolation of neonatal rat primary ventricular cells

Cardiomyocytes and ventricular fibroblasts were isolated from the same one-day-old Wistar rats (Wistar Rattus norvegicus) and in the same digestion solution, as previously described^29,30^. Briefly, the collected hearts were minced and digested with a collagenase II (126 U/ml) and pancreatin (0.2 mg/ml) solution. After six digestions, the pellet was filtered and pre-plated for 45 min at 37 °C under 5% CO_2_ in order to separate cardiac fibroblasts from cardiomyocytes. Cells were counted and plated in laminin-coated plates with a mixture of DMEM-Low and 199 media (4:1) containing 10% Horse Serum (HRS), 5% Newborn Calf Serum (NBCS), 1% penicillin–streptomycin, and 1% Bromodeoxyuridine (BrdU, Sigma B5002). Cardiomyocyte-enriched cultures were obtained by re-plating the supernatant of the previous incubation. Fibroblasts in passage 0 were kept in culture for a maximum of one week or before reaching confluence prior to plating for experiments (passage 1), in order to avoid differentiation into myofibroblasts. Cardiomyocyte-fibroblast co-cultures were obtained by adding to the cardiomyocyte-enriched supernatant previously isolated cardiac fibroblasts in a 1:1 proportion. The cell plating density was 1 × 10^5^ cells/cm^2^ for all culturing conditions. After 48 h the media was replaced every other day with DMEM-Low and 199 media (4:1) containing 1% penicillin–streptomycin, and 1% BrdU.

### Isolation of rat mesenchymal stem cells from adipose tissue

Mesenchymal stem cells were isolated as previously described^31^. Adult rat adipose tissue was minced into less than 3 mm^3^ pieces and washed with PBS. To isolate MSCs, the samples were centrifuged at 2000 rpm for 5 min to collect the fat tissue pieces. The fat pieces were then treated with type I collagenase. Enzyme activity was neutralized with DMEM high or low glucose containing 10% fetal bovine serum (FBS) and centrifuged at 1200 rpm for ten minutes to obtain a pellet. The pellet was re-suspended in complete medium (DMEM low containing 10% FBS, 1% penicillin–streptomycin) and filtered to remove the cellular debris. Cells were plated and allowed to adhere to a plastic culture dish and incubated overnight at 37 °C under 5% CO_2_. After 24 h contaminating blood cells that did not adhere were removed by changing the media. Mesenchymal stem cells were expanded and cryopreserved.

### Conditioned media

Cardiac fibroblast and MSC-conditioned media were obtained as previously reported^32^. Briefly, monolayers of either cardiac fibroblasts or MSCs were washed twice with PBS and incubated for 24 h at 37 °C under 5% CO_2_ with the control media (DMEM-Low + 199 media (4:1), containing 1% penicillin–streptomycin and 1% BrdU). The resultant conditioned media was collected and filtered with a 0.22 mm filter to remove cellular debris. Prior to use, the conditioned media was concentrated 10X using a centrifugal filter device with a 3,000 MW cutoff (Millipore, Burlington, USA), and then diluted to 1× with the control media, to exclude any possible influence of the previous consumption of the media by cardiac fibroblasts. Cardiomyocyte-enriched cultures received fresh conditioned media at 48 h intervals, always in parallel to the control group.

### Delimited co-culture system

The influence of microenvironment disposition of cardiac fibroblasts and cardiomyocytes was evaluated by a delimited co-culture system. We developed a cell barrier using the base of 1000 μl pipette tips. The cell barrier was placed onto the center of laminin-coated glass coverslips (Corning, Cat No. 2845-25) prior treated with ethanol 70% for 30 min. Cardiomyocytes were plated inside the cell barrier and cardiac fibroblasts outside. Cells were incubated for 24 h to adhere and then the cell barrier was removed to allow cells to form a contact zone. We obtained a delimited monolayer co-culture system consisting of a cardiomyocyte-enriched area, a contact zone and a cardiac fibroblast-enriched area. Cells on coverslips were cultivated in a higher volume of control media to highlight the influence of cell disposition in the microenvironment. The same co-culture system using cardiomyocytes on both sides, and MSCs outside instead of cardiac fibroblasts were used as controls. The functional and molecular analyses were done 7 days after plating.

### Quantitative characterization of cell cultures by flow cytometry

Flow cytometry was performed to analyze the cellular composition of neonatal rat primary ventricular cells in our cardiomyocyte-enriched cultures and cardiomyocyte-cardiac fibroblast mixed co-cultures at different times^33^. Samples were collected seven days after plating the cells. Antibodies to the phenotypic markers CD90 and CD31 were used to label, respectively, cardiac fibroblasts and endothelial cells. Cells negative for CD90 and CD31 were assigned as cardiomyocytes. Antibody staining was performed as described by each manufacturer.

### Relative gene expression analysis by RT-PCR

Total RNA was extracted with Trizol reagent according to manufacturer’s instructions. The real-time Reverse Transcription Polymerase Chain Reaction (RT-PCR) was performed as previously described^29^ to determine the relative gene expression of the transcription factors *Nkx2.5*, *Gata-4*, *Irx3*; Gap junction proteins connexin 43 (*Gja1*), connexin 40 (*Gja5*), and connexin 45 (*Gjc1*); and calcium channel subunit *Scn5a*. Cyclophilin was used as housekeeping gene and the results are disposed as 2^−ΔΔCt^.

### Immunofluorescence

Immunofluorescence assay was performed as previously described^34^ for high content screening analysis (IN Cell Analyzer 2200, GE) and confocal microscopy (Multiphoton Microscope LSM 780 NLO, Zeiss). Cells were fixed with 4% paraformaldehyde (PFA) for 60 min at room temperature, washed twice with PBS, permeabilized with 0.1% Triton for 15 min, and blocked with 5% Bovine Serum Albumin (BSA). Primary antibodies against the following proteins were used: 1:400 cardiac troponin I (4T21/2, HyTest), 1:200 cardiac troponin T (MA512960, Thermo Fisher), 1:900 vimentin (AB92547, Abcam), 1:750 connexin 43 (AB11370, Abcam), 1:100 connexin 40 (CX-40A, Alpha diagnostic International), 1:100 contactin-2 (AF4439, R&D Systems), 1:100 NAV1.5/*Scn5a* (sc-271255, Santa Cruz), and 1:200 ki67 (AB16667, Abcam). All primary antibodies were diluted in 2% BSA and incubated overnight at 4 °C. For the high content screening analysis, ten fields of each sample were collected and analyzed by the software IN Carta (GE Healthcare Life Sciences). The high throughput analysis retrieved phenotypic information of more than 500 single-cells per sample.

### Single-cell analysis of calcium transient and electrical properties assessed by patch-clamp technique

#### Calcium transient

Calcium transient recording experiments were performed as previously described^29^. In brief, experimental cells (cardiomyocytes, fibroblasts and mesenchymal stem cells) were plated on laminin-coated glass coverslips disposed as described in Method 2.5, inside P60 culture plates and analyzed 7 days after plating. After that, cells were changed to a fresh medium supplemented with 3 μM Fura-2 AM (F1201) and incubated for 15 min at 37 °C. The medium was refreshed once more with a medium free of Fura-2 AM and the coverslip transferred to a recording chamber. All the recordings were performed under electrical stimulation at 0.5 Hz, 4 ms and 10 V (Myopacer, IonOptix).

Fluorescence emission was 340–380 nm, and it was collected with a photomultiplier tube via the × 40 oil objective during continuous excitation at 510 nm with a 75-W Xenon lamp. We used the IonOptix Contractility System to record calcium transients. The recordings were assessed by an expert blinded to the experimental groups. Data were processed and analyzed using a customized homemade script built in MATLAB (MathWorks, Natick, MA). In brief, the raw traces were treated to erase technical deviations (e.g., alteration in the image focus that changed the trace to a different baseline). Also, the raw traces were filtered to reduce noise (sgolayfilt function in MATLAB). After filtering, representative peaks of each cell were selected, and different parameters were measured in the CaT (Amplitude, Time to peak, CaD50, CaD90, Ca decay, and resting period) as shown in Fig. 2. Fluorescence signals were corrected by their background and expressed as F/F0 (the ratio of fluorescence during the calcium transient divided by relaxation fluorescence). Mean curves of CaT of each experimental group were created by aligning the fluorescence of each cell recorded and calculated by the average of this fluorescence per time point, as illustrated in Figure Supplement 3.

CaT results were expressed as a mean ± SD to represent data average and variance. Each CaT parameter measured was previously subjected to the Anderson–Darling test to find the distribution type (Gaussian or Non-Gaussian). Gaussian distributed data were subjected to Parametric One-way-ANOVA and Non-Gaussian distributed data were subjected to Non-parametric Kruskal–Wallis test (both considered significant when p < 0.05). A Bonferroni *post-hoc* test was applied (alpha 0.05) to multiple comparisons on both parametric and non-parametric data.

#### Cellular electrophysiology

Whole-cell patch clamp technique was performed to record the action potential (AP) of our 7-day cultured cardiomyocytes’. For these experiments, cardiomyocytes were replated onto coverslips and APs were acquired after 24–48 h, at room temperature (22–25 °C) and using an EPC-10 patch clamp amplifier (HEKA, Holliston, Massachusetts, USA) in current clamp mode. Patch pippetes were pulled with tip resistance set at 2–2.5 MΩ and filled with and internal solution containing in mM: 20 KCl; 130 aspartic acid; 130 KOH; 10 HEPES; 2 MgCl_2_; 5 NaCl, pH 7.2. We used Tyrode as external solution (composed in mM: 140 NaCl, 5.4 KCl, 0.5 MgCl_2_, 0.33 NaH_2_PO_4_, 11 Glucose, 5 HEPES and 1.8 CaCl_2_, pH adjusted to 7.4). After establishment of the Whole-cell configuration, the amplifier was immediately switched to current-clamp mode and the cellular resting membrane potential was recorded. After 2–3 min stabilization between pipette solution and intracellular media was, APs were elicited by a square, depolarizing current pulses (2–4 ms duration and 300–500pA amplitude), at 1 Hz frequency. The AP threshold was recorded by a 5 ms current pulse with 25pA increment at 1 Hz frequency. All records were digitized at 10 kHz sampling rate.

### Preparation of cell lysate and Western blotting analysis

Cells were lysed in a SDS sample buffer (2% SDS, 5% glycerol, 60 mM Tris pH 6.8). The protein concentration was measured with a BCA protein assay kit (Pierce Biotechnology, Rockford, IL, USA)^35^. The following primary antibodies were used: Connexin 43 (ab11370, Abcam Cambridge, MA, USA), Connexin 40 (ab1726, Millipore Burlington, Ma, USA), Notch1 and Anti-activated Notch1 (ab8925, Abcam). Membranes were also probed with a monoclonal GAPDH-specific antibody (Abcam) as an internal control. After incubation for 1 h, at room temperature, with a horseradish peroxidase-conjugated immunoglobulin secondary antibody, the bound antibody was detected using an enhanced chemiluminescence system (GE Healthcare) according to the manufacturer's protocols. The signals were captured using a chemiluminescent image analyzer (ImageQuant LAS 4000 mini, GE HealthCare) and were quantified by densitometry using the ImageJ software (Wayne Rasband, National Institute of Health, USA).

### Neonatal rat ventricular cardiomyocyte organoid preparation

The organoids were prepared with the cardiomyocyte-enriched suspension obtained from the primary ventricular cells isolate using the 96-Well Bioprinting Kit (Greiner Bio-One Gmbh, 655840) according to the manufacturer’s protocol for cells in suspension. Briefly, we added 0.1 μL NanoShuttle/1 × 10^4^ cells. The cells and NanoShuttle mixture was gently ressuspended by pipetting and centrifuged at 1200 rpm for 5 min. The previous procedure was repeated three times before plating 5 × 10^4^ cells on a repellent 96-well plate (CELLSTAR Cell-Repellent, 650,970). The plate was placed onto de spheroid driver over night at 37 °C. The media was changed every 48 h and the cells were cultured for seven days. The LIVE/DEAD Viability/Cytotoxicity Assay Kit (ThermoFisher) was used according to the manufacturer’s instructions to assess organoid cell viability.

### DAPT treatment

Cells were treated with DAPT (Sigma-Aldrich, D5942-5MG) to test the role of the Notch pathway in cardiomyocytes and fibroblasts during the fast conduction fiber induction process. DAPT was diluted in DMSO and stored in 4 °C prior to use. We subjected the cardiomyocytes to different treatments: control media, fibroblast-conditioned media, conditioned media of fibroblasts previously inhibited by DAPT, and fibroblast-conditioned media with the concomitant addition of DAPT inhibitor (as described in Fig. 6A). DAPT was added to the media at a final concentration of 10 µM. Cardiomyocytes receiving DAPT had their media refreshed every 48 h for seven days. To obtain conditioned media of fibroblasts previously inhibited by DAPT, fibroblasts were treated with DAPT for 24 h, washed twice with PBS and then the standard procedure for obtaining their conditioned media was followed.

### Statistical analysis

Results were presented as a mean ± standard error of the mean (SEM). Statistical analyses were performed using the *one-way* ANOVA method with the Bonferroni *posthoc test*, unless stated otherwise. When appropriate, the results were analyzed by unpaired Student's t-test. Values of p < 0.05 were considered significant.

## Results

### Cardiac fibroblasts induce newborn rat cardiomyocyte expression of VCS markers in the adjacent microenvironment

**Figure 1:**
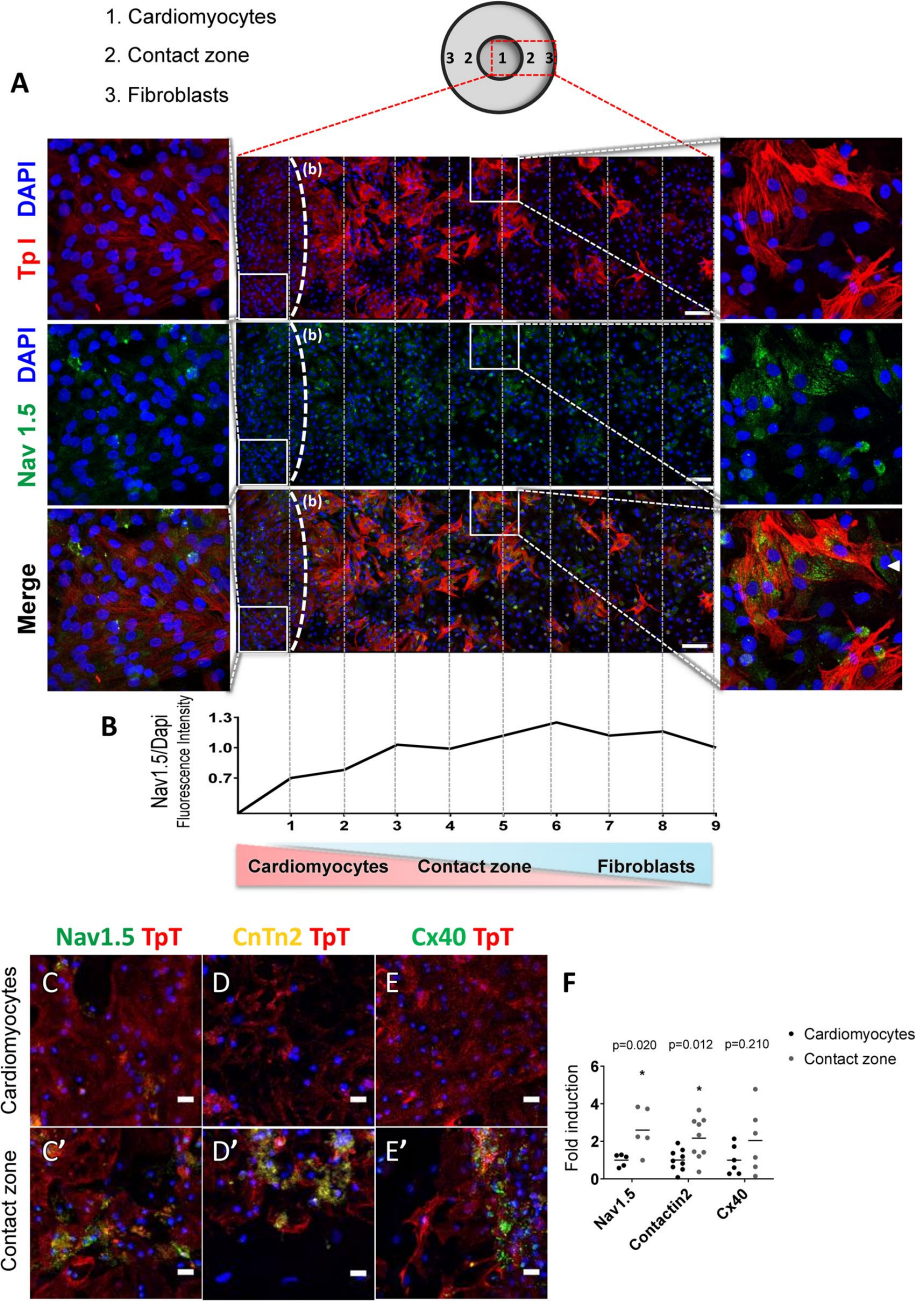
Cardiac fibroblasts induce the expression of VCS markers in adjacent cardiomyocytes. (**A**) Representative image of the cardiomyocyte-fibroblast delimited co-culture. Cell nuclei (DAPI), cardiomyocytes (cardiac Troponin I positive cells, Red), cardiac fibroblasts (cardiac Troponin I negative cells), and Nav1.5 (Green). The dotted line (b) delimits the cardiomyocyte-enriched area on the left side from the beginning of the contact zone. Samples of the cardiomyocyte-enriched area and the contact zone are magnified at the left and right side of the figure, respectively. Arrowhead in the magnified contact zone highlights a fibroblast expressing Nav1.5. Scale bars = 100 µm. (**B**) Fluorescence intensity of Nav1.5/Dapi. (**C**–**E**; **C’**–**E’**; **F**) Representative images and quantification of the protein expression of the VCS markers NAV1.5, Contactin-2 and Connexin 40 in the cardiomyocyte-enriched area (cardio) and the contact zone (Contact) of the same co-cultures. The relative protein expression in the contact zone was defined as the fold induction of the cardiomyocyte-enriched area from the same cover slip. Results are disposed as a mean ± SEM. Data were subjected to paired Student’s t-test against the cardio group; p < 0.05 was considered significant. N = 6–12 (four different experiments). Scale bars = 20 µm.

To investigate whether cardiac fibroblasts trigger neighboring cardiomyocyte specialization into the conduction phenotype, we developed a monolayer co-culture consisting of a cardiomyocyte-enriched area, followed by a contact zone and a fibroblast-enriched area (Fig. 1). We isolated primary ventricular cells from one-day-old neonatal rats and pre-plated them to separate cardiomyocytes from fibroblasts. To ensure that we were working with cardiac fibroblasts and not myofibroblasts, we made an immunofluorescence panel with specific markers for fibroblasts (Figure S1). Analysis of fibroblast vs. myofibroblast markers was performed in fibroblast cultures seven day after plating and followed the same experimental conditions undertaken throughout this study. The fibroblast cultures were positive for vimentin, fibronectin, CD90 and PDGFRα, and negative for αSMA (smooth muscle alpha actin). Additionally, collagen synthesis and cross-linking was less apparent in our fibroblast cultures compared to myofibroblasts (Figure S1B). Altogether, these data imply that fibroblasts were not differentiated into myofibroblasts under the experimental conditions.

Immunostaining of the VCS markers NAV1.5, CONTACTIN-2 and CONNEXIN-40 was used to identify VCS-like cells^8,36,37^. Cardiomyocytes were distinguished from cardiac fibroblasts by troponin I positive staining. Clusters of cardiomyocytes with enriched expression of NAV1.5 appeared in the contact zone with cardiac fibroblasts, but were only sparsely found within the cardiomyocyte-enriched area (Fig. 1A). The qualitative comparison of the contact zone and cardiomyocyte-enriched area (enlarged respectively on the right and left side of Fig. 1A) shows NAV1.5 positive cardiomyocytes in the contact zone, and the weak labeling in the cardiomyocyte-enriched area, which was also confirmed by the longitudinal quantification of NAV1.5 intensity (Fig. 1B). A few fibroblasts expressed NAV1.5, as indicated by the arrowhead in the magnified contact zone (Fig. 1A). Fibroblasts were reported to express low amounts of NAV1.5 and the increased expression of this channel was related to fibroblast differentiation^38^. Low or no expression of NAV1.5 in our co-cultures, together with the negative staining for αSMA (Figure S1), indicate that our fibroblasts were undifferentiated.

The VCS markers NAV1.5 and CONTACTIN 2 displayed enriched expression by two-fold in the contact zone compared to the cardiomyocyte-enriched area (1.00 ± 0.15 vs. 2.60 ± 0.53; p = 0.02; and 1.00 ± 0.19 vs. 2.17 ± 0.37; p = 0.012; Fig. 1C,D,C’,D’,F), while the expression of CONNEXIN 40 showed no difference between contact zone and cardiomyocyte-enriched area (1.00 ± 0.32 vs. 2.04 ± 0.71; p = 0.21; Fig. 1E,E’,F). We also observed a two-fold enrichment of Troponin T expression in cardiomyocytes located in the contact zone (data not shown). Taken together, these data suggest that cardiac fibroblasts induce the expression of VCS markers in adjacent newborn rat cardiomyocytes.

### Cardiomyocytes adjacent to cardiac fibroblasts display a VCS-like CaT profile

To verify whether the expression of VCS markers in cardiomyocytes adjacent to cardiac fibroblasts was accompanied by functional changes corresponding to fast-conducting cells, we analyzed cardiomyocyte intracellular calcium transients (CaTs) in the delimited co-culture system previously described (Fig. 1). Moreover, a co-culture with mesenchymal stem cells (MSCs) rather than cardiac fibroblasts, and a cardiomyocyte-only culture was used as controls to assess the specificity of the cardiomyocyte-cardiac fibroblast interaction.

**Figure 2:**
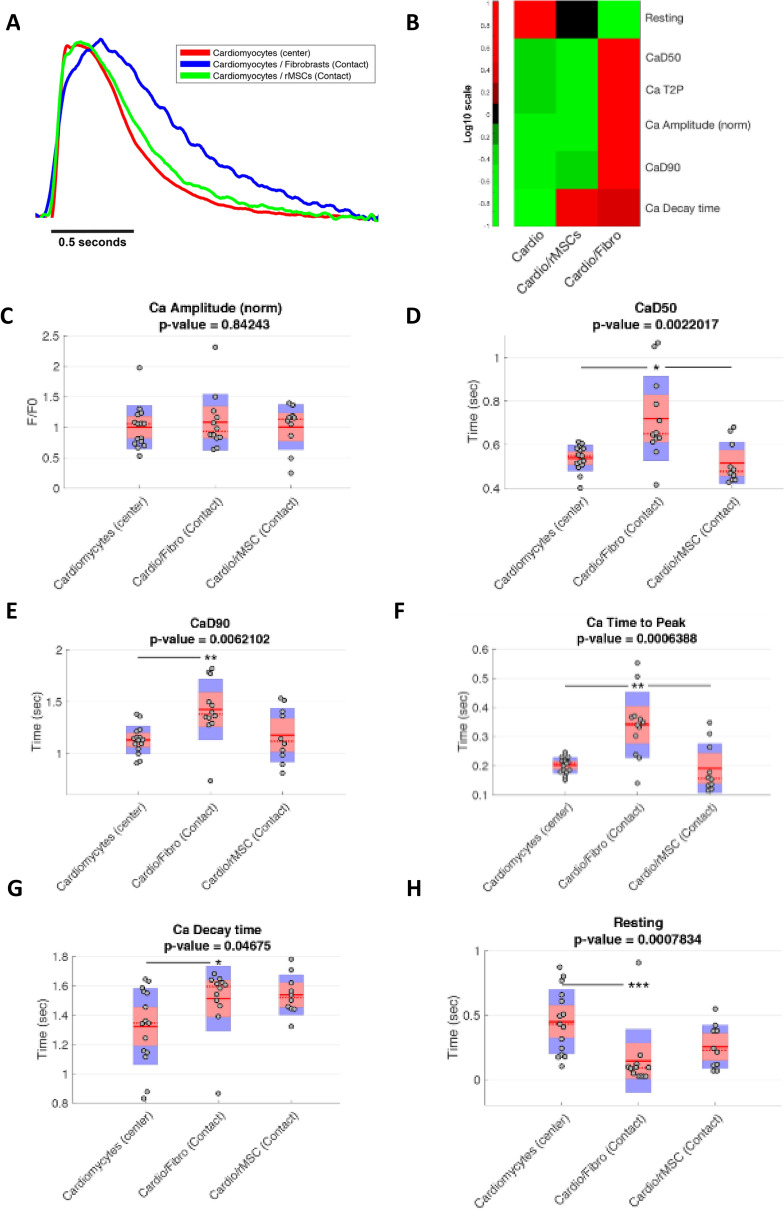
Cardiac fibroblasts influence calcium handling of adjacent cardiomyocytes. Cardiomyocytes were plated in the center of the delimited co-culture system and either cardiac fibroblasts or mesenchymal stem cells were plated on the outside area. The calcium transients of contractile cells in the contact zones and cardiomyocyte-enriched areas were recorded and measured. (**A**) CaT mean curves for each experimental group. (**B**) Heatmap analysis of CaT parameters for each experimental group. (**C**) Normalized CaT amplitudes. (**D**) Time to reach 50% of calcium decay (CaD50). (**E**) Time to reach 90% of calcium decay (CaD90). (**F**) Time to reach the maximum peak of calcium per beating cycle (Time to Peak). (**G**) Time to reach basal levels of Calcium after maximum peak (Ca decay time). (**H**) The Resting interval or the time between the basal level reached after contraction until the new CaT. The cardiomyocyte-enriched area in the center of the cardiomyocyte-only coverslip was used as the control. p < 0.05, **p < 0.01, ***p < 0.001 after Bonferroni *post-hoc* test (alpha 0.05). Sample sizes were 15 cells of Cardiomyocytes (center), 12 cells of Cardio/Fibro (contact) and 10 cells of Cardio/MSCs (contact) from three different experiments.

The global analysis of the mean calcium transient (CaT) curves showed that cardiomyocytes in contact with cardiac fibroblasts had longer CaTs, while cardiomyocytes in contact with MSCs did not differ from the control cardiomyocyte-only group (Fig. 2A,B).

**Figure 3:**
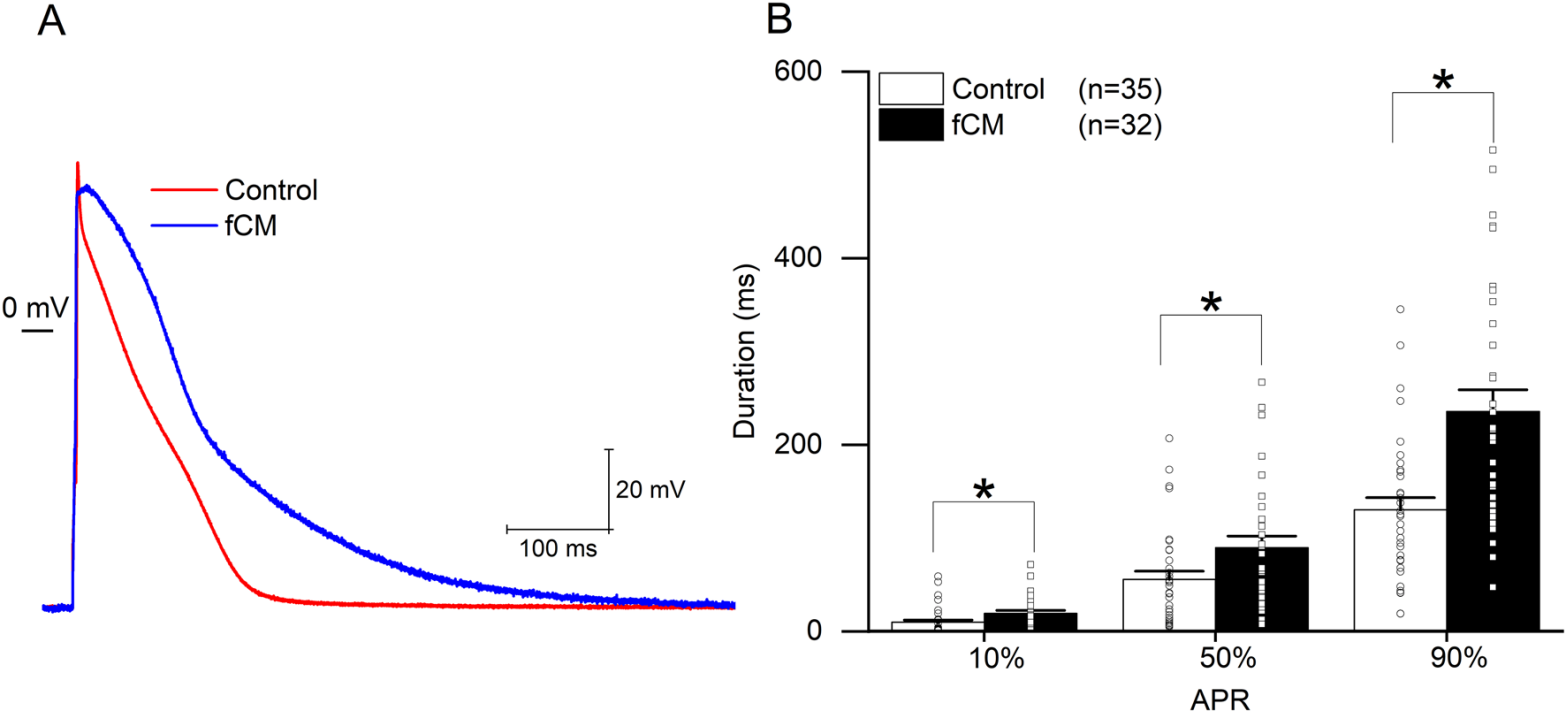
Electrical properties of cardiomyocytes exposed to fibroblast conditioned media (fCM). (**A**) Representative traces of action potential in control (red) and fCM exposed cardiomyocytes (blue) groups. (**B**) Time required to reach 10%, 50%, and 90% of full AP repolarization. APR represents action potential repolarization, and n the number of cells. *p < .05 using Two-sample t-Test.

Further analysis showed that CaT amplitudes did not differ in contact zones between different groups (Fig. 2C). The time to reach 50% and 90% of calcium decay (CaD50 and the CaD90) were approximately 34% and 26% longer in cardiomyocytes in contact with cardiac fibroblasts than in the cardiomyocytes alone (0.72 ± 0.185 s vs. 0.54 ± 0.057 s; p < 0.05; Fig. 2D and 1.42 ± 0.281 s vs. 1.13 ± 0.130 s; p < 0.01; Fig. 2D,E). The time to peak was over 60% slower in cardiomyocytes in contact with cardiac fibroblasts compared to the control (0.34 ± 0.108 s vs. 0.201 ± 0.026 s; p < 0.01; Fig. 2F). Calcium decay time was almost 15% longer in cardiomyocytes in contact with cardiac fibroblasts (1.51 ± 0.212 s vs. 1.32 ± 0.251 s; p < 0.01; Fig. 2G). The resting interval, the time between the first second after baseline calcium recovery and the start of a new CaT, was 68% shorter in cardiomyocytes in contact with cardiac fibroblasts compared with the control (0.14 ± 0.235 s. vs. 0.45 ± 0.242 s; p < 0.001; Fig. 2H). CaTs did not show consistent differences between cardiomyocyte-enriched areas in the two different co-cultures, cardiac fibroblasts or MSCs, and the cardiomyocyte-only group (Figure S2). We then used patch-clamp technique to better understand the excitable properties of the cells. Cell capacitance and threshold were similar between fCM and control cells (45.2 ± 49 pF (n = 37) vs. 32.3 ± 2.8 pF (n = 42) (p = 0.09), respectively) and (243.8 ± 26.6, n = 28 vs. 191.1 ± 17.5 pA, n = 25 (p > 0.05), respectively). However, we observed a longer action potential in fCM compared to control cells as indicated by the increased time to reach 10, 50 and 90% of repolarization (Fig. 3). Moreover, despite the fact that the action potential amplitude and resting membrane potential (RMP) were comparable between the fCM and control cells (99.4 ± 2.6 vs. 100.40 ± 2.8 mV (p > 0.05)) and (− 67.6 ± 0.7 vs. − 68.9 ± 1.1 mV (p > 0.05), respectively), we observed a decrease in the maximal rise of the slope velocity (dV/dt) of the action potential in fCM compared to control cells (88.9 ± 6.9 vs. 135.9 ± 12.1 mV/ms (p < 0.05), respectively).

In summary, analyses of individual parameters support that cardiomyocytes neighboring cardiac fibroblasts display CaTs similar to rapidly conducting fibers, while the same is not observed in cardiomyocytes in the contact zone with MSCs. Similarly, cardiomyocyte exposed to fibroblast conditioned media displayed properties of VCS-like cells evaluated by patch-clamp. Altogether, these functioning findings agree with the data shown above indicating that these cells also display increased expression of VCS markers.

### Cardiac fibroblast-conditioned media is sufficient to activate the VCS gene network in cardiomyocytes

To investigate whether the molecular and functional changes towards the fast-conducting phenotype are caused by an alteration of cardiomyocyte transcriptional signature and gene network profile, we evaluated the gene expression of determining VCS transcription factors and downstream VCS genes in mixed co-cultures of cardiomyocytes and cardiac fibroblasts.

**Figure 4:**
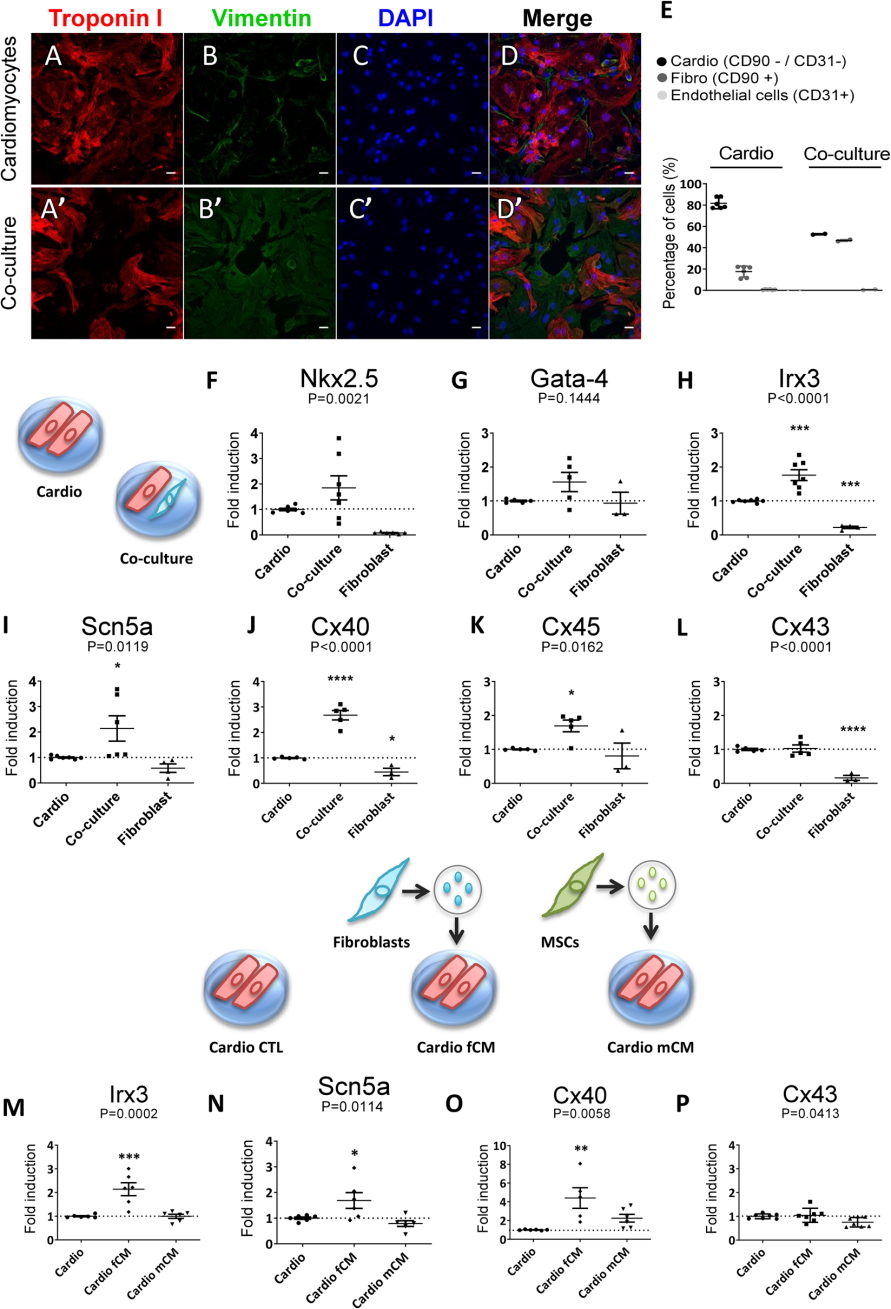
The paracrine effect of fibroblast-secreted mediators is sufficient to regulate key genes of the VCS genetic program. (**A**–**D**;**A**’–**D**’) Immunofluorescence imaging of cardiomyocyte-enriched cultures and co-cultures seven days after plating. Cell nuclei (DAPI), cardiomyocytes (cardiac Troponin I positive cells, Red), and cardiac fibroblasts (Vimentin positive cells, Green). Scale bars = 20 µm. (**E**) Cellular composition of cardiomyocyte-enriched cultures and co-cultures by flow cytometry. N = 3–5. (**F**–**P**) Relative expression of key cardiac genes determined by real time RT-PCR seven days after plating. (**F**–**L**) Total RNA was isolated from whole extracts of cardiomyocyte-enriched cultures, cardiomyocyte-cardiac fibroblast co-cultures, and cardiac fibroblast cultures. After fold induction calculation, relative gene expression was normalized by the number of cardiomyocytes to avoid bleaching effects due to the augmented proportion of cardiac fibroblasts in the co-cultures. (**M**–**P**) Total RNA was isolated from whole extracts of cardiomyocyte-enriched cultures treated with control, cardiac fibroblast- (fCM) or MSC-conditioned media (mCM)*.* Cyclophilin and GAPDH were used as housekeeping genes and the results are displayed as 2^-ΔΔCt^ , mean ± SEM. Data were subjected to one-way ANOVA, followed by multiple comparisons against the cardiomyocyte-enriched culture group, used as control; p < 0.05 was considered significant. N = 3–7 (three different experiments).

Our experiments were performed using co-cultures with 50% cardiomyocytes and cardiac fibroblasts, and cardiomyocyte-enriched cultures consisting of 80% of cardiomyocytes seven days after plating (Fig. 4A–E). Cells were maintained in culture media containing BrdU to inhibit excessive fibroblast proliferation. After determining the gene fold induction, we normalized the expression data of mixed co-cultures and cardiomyocyte cultures to the respective number of cardiomyocytes.

The gene expression of the transcription factors *Nkx2.5* and *Irx3* was increased in the co-cultures compared to the control group, corresponding to the cardiomyocyte-enriched cultures (1.00 ± 0.05 vs. 1.85 ± 0.47 p = 0.0021; Fig. 4F) and (1.00 ± 0.02 vs. 1.76 ± 0.16 p = 0.0001; Fig. 4H). On the other hand, increased *Gata-4* gene expression in the co-cultures was not statistically significant (1.00 ± 0.02 vs. 1.56 ± 0.28 p = 0.1444; Fig. 4G). These transcription factors are key modulators of cardiomyocyte fate and postnatal maturation into fast-conducting fibers and up-regulate the VCS gene network^10,12,16,18,39^. We then evaluated the expression of downstream genes related to the VCS network. The gene expression of the sodium channel subunit NAV1.5, encoded by the *Scn5a* gene, was significantly increased in the co-cultures compared to the control (1.00 ± 0.03 vs. 2.14 ± 0.49; p = 0.0119; Fig. 4I). Connexins 40 and 45, which are mainly expressed in the cardiac conduction system, also displayed increased gene expression in co-cultures (1.00 ± 0.02 vs. 2.68 ± 0.18; p < 0.0001; and 1.00 ± 0.01 vs. 1.69 ± 0.17; p = 0.0162; respectively, Fig. 4J,K), but connexin 43 remained unchanged (1.00 ± 0.03 vs. 1.02 ± 0.11; ns; Fig. 4L), which is mainly expressed in working cardiomyocytes. Therefore, these data support a specific shift of cardiomyocyte transcriptional signature to the conduction profile.

To investigate whether the fibroblast-mediated transcriptional activation of VCS genes in cardiomyocytes was caused by signaling of transmembrane proteins or soluble mediators secreted by cardiac fibroblasts, we compared cardiomyocyte-enriched cultures with the control media and cardiomyocyte-enriched cultures that received either conditioned media by cardiac fibroblasts (fCM) or by mesenchymal stem cells (mCM).

Cardiac fibroblast-conditioned media was sufficient to induce a two-fold increase in the gene expression of *Irx3* in cardiomyocytes treated with fCM compared to the control (1.00 ± 0.03 vs. 2.14 ± 0.27; p = 0.0002; Fig. 4M). The gene expression of *Scn5a* and connexin 40 were also significantly increased in cardiomyocytes that received fCM (1.00 ± 0.04 vs. 1.69 ± 0.30; p = 0.0114; Fig. 4N) and (1.00 ± 0.02 vs. 4.40 ± 1.10; p = 0.0058; Fig. 4O), while the expression of connexin 43 remained unchanged in all groups (1.00 ± 0.04 vs. 1.04 ± 0.11 vs. 0.75 ± 0.07; ns; Fig. 4P). The mesenchymal stem cell conditioned media (mCM) did not cause any alteration in the gene expression of the VCS markers nor in connexin 43, despite the similarity of MSCs with fibroblasts, which emphasizes the specific effects by fibroblasts. To exclude the possibility that the observed alterations in gene expression were due to differential cell proliferation, we assessed proliferating KI67 positive cells by high content screening. The percentages of proliferating cardiomyocytes (cardiac troponin I + KI67 + cells) in the co-culture and in the fCM group did not differ from the control (9.39 ± 1.16 vs. 11.39 ± 0.90; ns; and 9.39 ± 1.16 vs. 9.20 ± 0.57; ns; Respectively, Figure S5I). On the other hand, cardiomyocyte-enriched cultures treated with mCM presented about 5% increase in cardiomyocyte proliferation (9.39 ± 1.16 vs. 14.34 ± 1.96; p = 0.0323; Figure S5I). The rates of proliferating cardiac fibroblasts (cardiac troponin I-KI67 + cells) remained unchanged among the analyzed groups compared to the control (Figure S5J). These results are consistent with the idea that conditioned media from fibroblasts is sufficient to induce the maturation of neonatal cardiac myocytes towards the VCS phenotype independently of cell proliferation. Altogether, these data suggest that cardiac fibroblasts induce the VCS-gene network in cardiomyocytes and that the soluble mediators secreted by cardiac fibroblasts are sufficient to recapitulate this response.

### Cardiac fibroblast-conditioned media reduces the number of working myocyte-like cells

**Figure 5:**
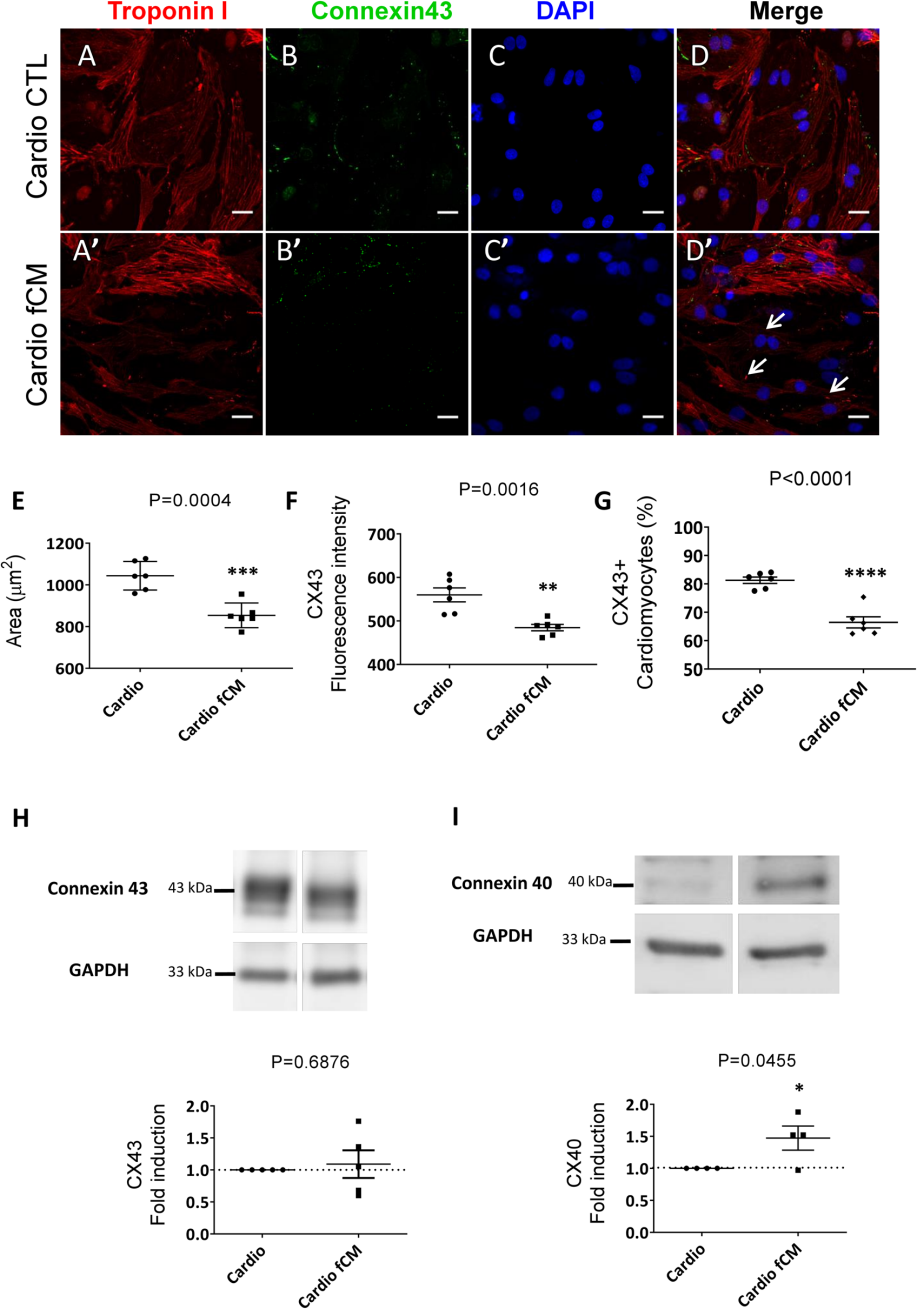
Cardiac fibroblast-conditioned media reduces the proportion of cells expressing working myocyte characteristics. Working myocyte phenotypic analysis by high content screening. Phenotypic parameters were retrieved for each single-cell identified and only cardiomyocytes were included in the analysis. (**A**–**D**’) Control and fCM-treated group representative immunofluorescence images, respectively. Cell nuclei were stained with (DAPI), cardiomyocytes (cardiac Troponin I positive cells, Red), and connexin 43 (Green). Asterisk symbol illustrates working myocyte expressing connexin 43. Arrows indicate smaller troponin I positive cells which do not express connexin 43. Scale bars = 20 µm. (**E**) Mean cardiomyocyte area in micrometers (μm^2^) given by the average of total troponin I area surrounding a valid nucleus. (**F**) Mean expression of connexin 43 per cardiomyocyte given by the average of connexin 43 fluorescence intensity in the cardiomyocyte population. (**G**) Percentage of cardiomyocytes connexin 43 positive (Cx43 +) in the cardiomyocyte population. Results are disposed as a mean ± SEM. Data were subjected to unpaired Student’s t-test against the control group; p < 0.05 was considered significant. N = 6 (each consisting of more than 500 cells). (**H**–**I**) Relative protein expression of connexin 40 and connexin 43 determined by Western Blotting. Gels were cut to keep the groups of interest together and full-length blots are presented in Supplementary Figure S6A-B. Protein samples were isolated from whole extracts of cardiomyocyte-enriched cultures treated with the control or cardiac fibroblast-conditioned media (fCM). GAPDH was used as housekeeping protein, and the results are disposed as the relative expression to the control group, mean ± SEM. Data were subjected to unpaired Student’s t test, against the control group; p < 0.05 was considered significant. N = 6.

We then evaluated whether the cardiac fibroblast-conditioned media in addition to activating cardiomyocyte specialization towards the ventricular conduction-like system also inhibited the working cardiomyocyte phenotype. We assessed phenotypic changes related to working cardiomyocytes at single cell level with the High Content Screening technique that analyzes large-scale immunofluorescence images (representative images, Fig. 5A–D’). Only cardiac troponin I positive cells corresponding to cardiomyocytes were considered for the analysis. The average area of cardiomyocytes decreased by more than 100 μm^2^ in the fCM group compared to the control group (1044 ± 28.03 μm^2^ vs. 853.90 ± 24.13 μm^2^; p = 0.0004; Fig. 5E), as shown in Fig. 5D’ where it is possible to observe smaller positive troponin I cells. Consistently, rat Purkinje fibers are smaller than ventricular working myocytes^40^.

Although there was no reduction in connexin 43 gene and protein expression levels in total extracts compared to the control (1.00 ± 0.04 vs. 1.04 ± 0.11 vs. 0.75 ± 0.07; ns; Fig. 4L) and (1.00 vs 1.09 ± 0.21; ns; Fig. 5H), the mean protein expression of connexin 43 by cardiomyocyte was reduced in the group receiving fCM (560 ± 16, 01 vs 484.7 ± 7.32; p = 0.0016; Fig. 5F). It is important to emphasize that we specifically quantified the expression of connexin 43 per individual cardiomyocyte, reducing the noise generated by the inclusion of other cell types that were present in the total extract. We evaluated whether this decrease in connexin 43 expression was due to lower expression by cardiomyocyte or a reduction in connexin 43 expressing cardiomyocytes in the fCM-treated group. In fact, in the fCM-treated group there was a reduction greater than 10% in connexin 43-expressing cardiomyocytes in the cardiomyocyte population (81.28 ± 1.12% vs. 66.43 ± 1.97%; p < 0.0001 Fig. 5G). Alternatively, connexin 40 gene and protein expression levels increased in fCM-treated cardiomyocytes compared to the control (1.00 ± 0.02 vs. 4.40 ± 1.10; p = 0.0058; Fig. 4O) and (1.00 vs. 1.47 ± 0.19; p < 0.05; Fig. 5I), respectively. Working cardiomyocytes are larger than fast-conducting fibers and express large amounts of connexin 43, while expression of connexin 40 is mostly restricted to the VCS^8,40^. Therefore, these data suggest that the fibroblast-conditioned media reduced the number of working cardiomyocytes and augmented the number of cardiomyocytes displaying VCS-like characteristics in the cell population.

### Fibroblast-secreted factors induce 3-dimensional up regulation of VCS markers

**Figure 6:**
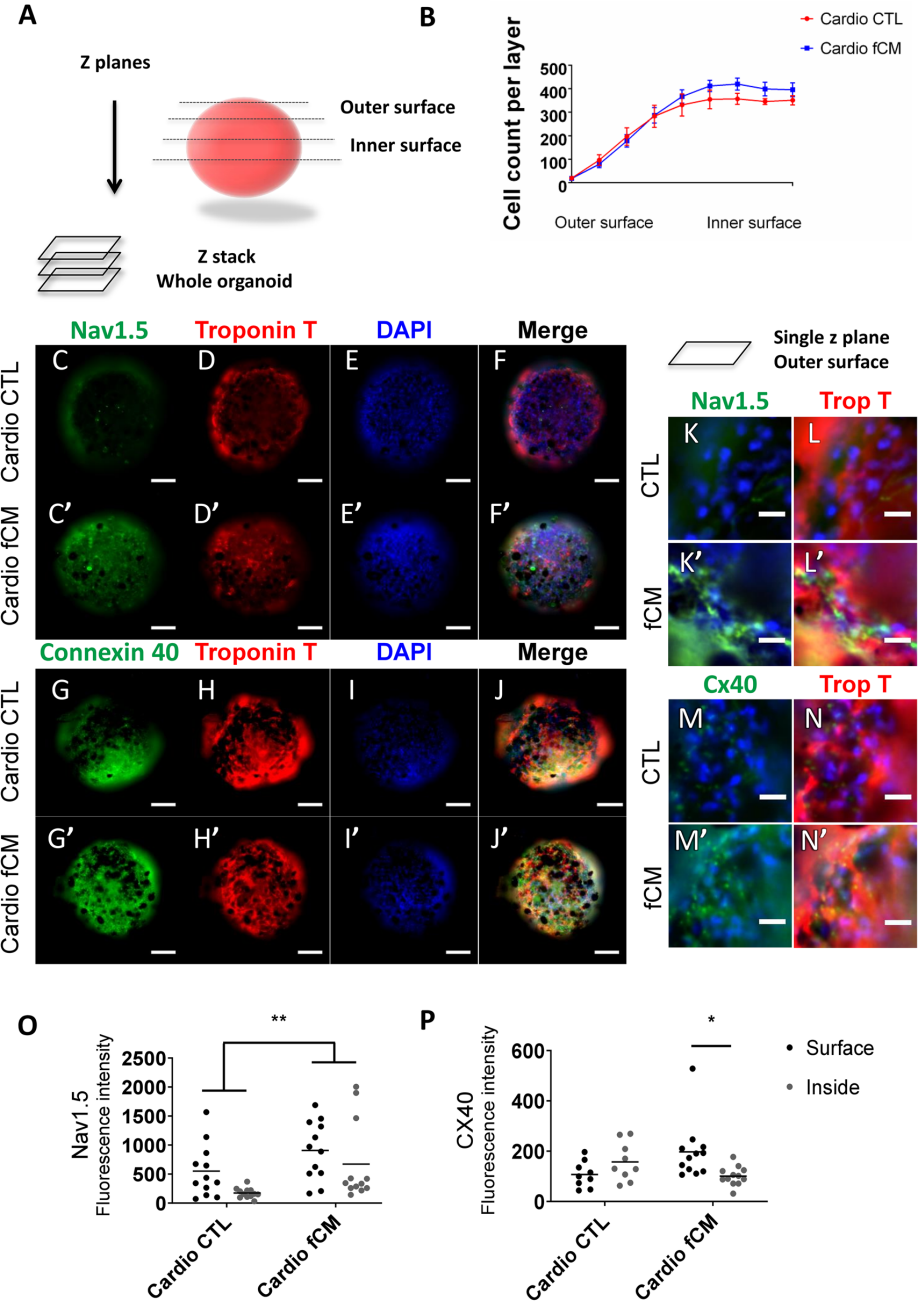
Fibroblast-secreted mediators induce 3-dimensional patterning of VCS markers. Evaluation of the influence of fibroblast-secreted factors on the expression of VCS markers in neonatal rat cardiomyocyte organoids using automated high content screening analysis. Phenotypic parameters were retrieved for each single-cell identified in 12 Z-planes and only cardiomyocytes were included in the analysis. (**A**) Illustration of cardiomyocyte organoid analysis (**B**) Total number of cells per z plane. N = 6–8. (**C**–**J**’) Control and fCM-treated organoids representative z-stack immunofluorescence images from summed intensities. Cell nuclei (DAPI), cardiomyocytes (cardiac Troponin T positive cells, Red), connexin 40 and NAV1.5 (Green). Scale bars = 100 µm. (**K**–**N**’) Single z plane view of representative outer surface regions of control and fCM-treated organoids. (**O**–**P**) Mean expression of connexin 40 and NAV1.5 per cardiomyocyte given by the average of fluorescence intensity in the cardiomyocyte population. The mean fluorescence intensity was measured in three different z-positions for the organoid outer (z4–z6) and inner surfaces (z8–z10). Results are disposed as a mean ± SEM. Data were subjected to a two-way ANOVA followed by a post test comparing each group to every other group; p < 0.05 was considered significant. N = 9–12.

In order to better understand the impact of cardiomyocyte disposition in the microenvironment for the induction of the VCS-like phenotype mediated by fibroblast-secreted factors, we cultured both cell types in an organoid structure and exposed them to either fibroblast-conditioned media or control media for seven days. We first confirmed the cell viability in the organoids as shown in Figure S4, since their feeding is limited by diffusion. Under the present conditions, we found no signs of cell death in the control organoids nor in the fCM treated group. We evaluated the 3-dimensional expression of VCS markers by immunofluorescence using high content screening analysis (representative images of the reconstructed z-stack as illustrated in Fig. 6A, and shown in Fig. 6C–J’, representative images of single z planes Fig. 6K–N’). The expression of NAV1.5 displayed a 60% and three-fold increase in the outer and inner surfaces of the fCM group compared to the control, respectively (Cardio CTL 550.2 ± 131.5 (Outer) and 173.3 ± 25.23 (Inner) vs. Cardio fCM 906.6 ± 145.7 (Outer) and 671.6 ± 199.1; Treatment P < 0.01; Fig. 6O). The organoids treated with fCM also displayed a two-fold increase in the expression of connexin 40 in the outer surface compared to its inner surface (197.4 ± 32.85 (Outer) vs. 100.9 ± 10.81 (Inner); p = 0.04; Fig. 6P), while the control organoid showed the reversed expression pattern (Cardio CTL 107.4 ± 17.25 (Outer) and 157.4 ± 25.54 (Inner) vs. Cardio fCM 197.4 ± 32.85 (Outer) and 100.9 ± 10.81 (Inner); Interaction p < 0.05; Fig. 6P) with higher connexin 40 expression in its inner surface, which was proportional to the number of cardiomyocytes in the correspondent layers (Fig. 6B). These data further support that fibroblast-secreted factors promote the expression of VCS markers in cardiomyocytes in a spatially dependent manner.

### Activation of the NOTCH1 pathway in cardiomyocytes and fibroblasts is required for cardiomyocyte maturation into VCS-like cells

**Figure 7:**
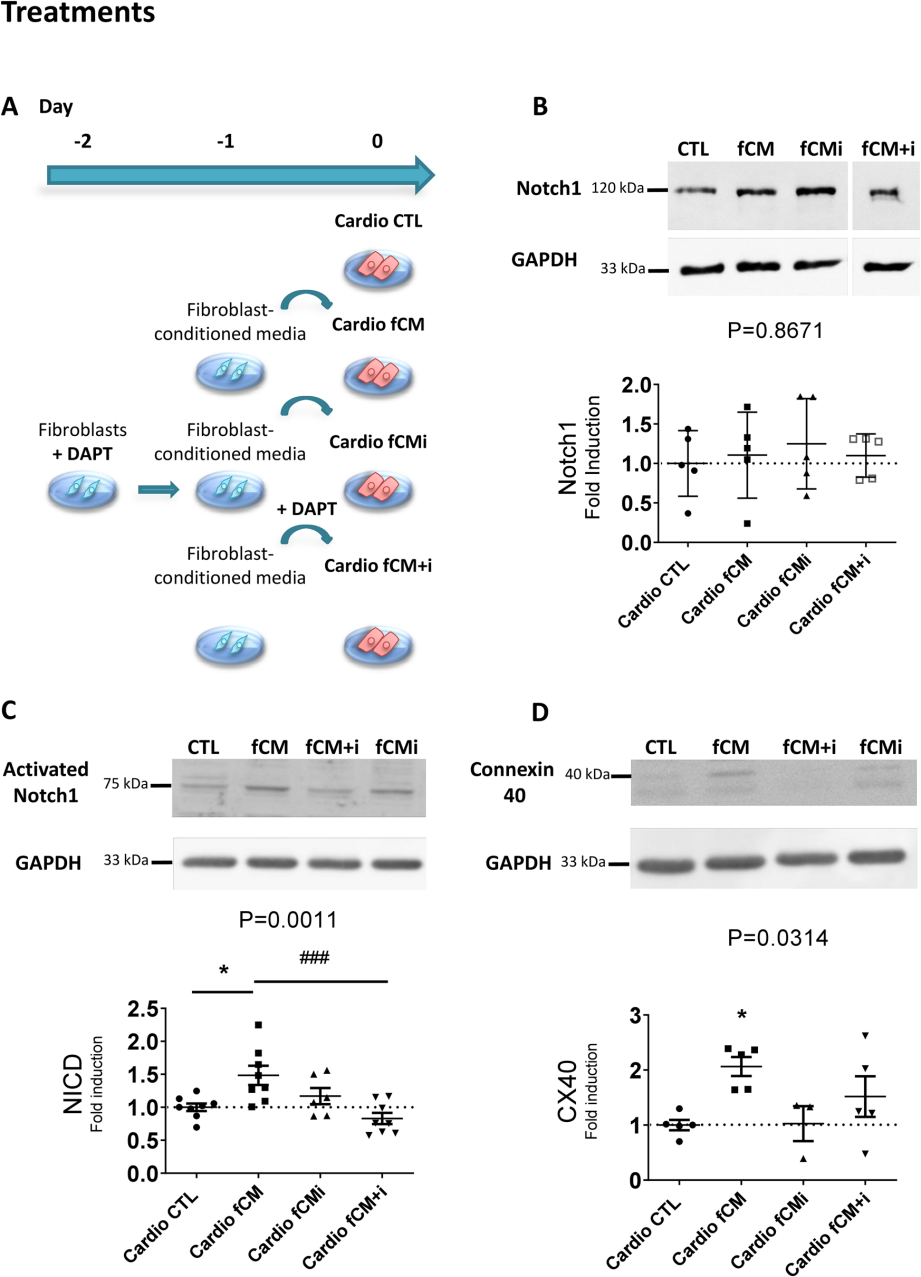
Notch1 pathway has a central role in the fCM-mediated induction VCS-like cells. Protein samples were isolated from total extracts of cardiomyocyte-enriched cultures treated with control media (CTL), fibroblast-conditioned media (fCM), conditioned media of fibroblasts previously inhibited with DAPT (fCMi) or fibroblast-conditioned media with the concomitant addition of DAPT inhibitor (fCM + i). Relative protein expression was analyzed by Western Blot. (**A**) Illustrative scheme of treatments. Each group was cultured for seven days and had its media changed every 48 h. (**B**) Relative protein expression levels of NOTCH1 receptor in cardiomyocytes according to the defined treatments, N = 5 Gel was cut to keep the groups of interest together and full-length blots are presented in Supplementary Figure S6C. (**C**) Relative protein expression levels of the activated-NOTCH1 receptor (NICD, *Notch IntraCellular Domain*) in cardiomyocytes. N = 6–8. (**D**) Relative protein expression levels of connexin 40 in cardiomyocytes according to treatments, N = 3–5. The GAPDH protein was used as housekeeping protein, and the results are disposed as the relative expression to the control group for each gel, mean ± SEM. Data were submitted to the one-way ANOVA test and Dunnett's posthoc test against the control group; p < 0.05 was considered significant.

We then assessed the role of the Notch pathway in the cardiac fibroblast-mediated induction of VCS-like cells. We used DAPT to inhibit NOTCH cleavage and its activation in cardiomyocytes and fibroblasts during the fast conduction fiber induction process. We subjected the cardiomyocytes to different treatments: control media, fibroblast-conditioned media, the conditioned media of fibroblasts previously inhibited by DAPT, and fibroblast-conditioned media with the concomitant addition of DAPT inhibitor (as described in Fig. 7A). Protein expression of total and activated NOTCH (NICD, Notch Intracellular Domain), as well as that of connexin 40, used as a marker for the conduction system, were evaluated by Western Blot.

NOTCH1 protein expression does not differ between groups (Fig. 7B), suggesting that fibroblast-conditioned media and DAPT do not influence NOTCH1 protein expression. However, fibroblast-conditioned media was sufficient to increase NOTCH1 active protein form in cardiomyocytes by approximately 50% (1.00 ± 0.058 vs. 1.48 ± 0.14; p = 0.0011; Fig. 7C), as well as to cause a two-fold increase in the expression of connexin 40 (1.00 ± 0.094 vs. 2.06 ± 0.17; p = 0.0314; Fig. 7D). By adding DAPT along with the fibroblast conditioned medium treatment, we observed a significant reduction of activated NOTCH1 compared to the conditioned medium alone, reaching values similar to the control group (1.48 ± 0.14 vs. 0.83 ± 0.08; p = 0.0011; Fig. 7C). Importantly, the increase in connexin 40 expression was prevented (1.00 ± 0.09 vs. 1.52 ± 0.36; p = ns; Fig. 7D), suggesting that NOTCH1 activation in cardiomyocytes is required for specialization in fast-conducting fibers in response to the factors secreted by fibroblasts. Moreover, inhibition of the NOTCH pathway with DAPT in fibroblasts resulted in the loss of the activating effect of the fibroblast-conditioned media over the NOTCH1 pathway and connexin 40 expression in cardiomyocytes, as demonstrated by the fCMi treatment (1.00 ± 0.058 vs. 1.17 ± 0.12; and 1.00 ± 0.09 vs. 1.02 ± 0.31; p = ns. Figure 7C,D). This response highlights the role of the activation of the NOTCH pathway also in fibroblasts for the production of the soluble mediators responsible for inducing the VCS phenotype in cardiomyocytes. Cardiomyocytes treated with DAPT did not present any alteration when compared to control cardiomyocytes (data not shown).

Together, these data support that NOTCH1 pathway activation is necessary for fibroblast secretion of factors that mediate cardiomyocyte specialization in VCS-like cells. Therefore, NOTCH1 appears as a central signaling pathway in the crosstalk between fibroblasts and cardiomyocytes for the induction of cardiomyocyte maturation into fast-conducting fibers.

## Discussion

Different studies have shown the importance of postnatal maturation for the ventricular conduction system and the murine newborn cardiomyocyte plasticity to differentiate into conduction cell in vivo and in vitro^10,12,24,39^. In this study, we provide molecular and functional evidence that cardiac fibroblasts secrete factors that trigger neonatal rat cardiomyocyte specialization into VCS-like cells in vitro*,* through NOTCH1 activation in both fibroblasts and cardiomyocytes.

Using a delimited co-culture system, we demonstrated that juxtacrine signaling between cardiac fibroblasts and cardiomyocytes was instrumental to induce the expression of VCS markers in cardiomyocytes. The protein expression of NAV1.5 and CONTACTIN 2 increased in the contact zone between cardiac fibroblasts and cardiomyocytes. In agreement with the expression of VCS markers, cardiomyocyte calcium transients in the contact zone were similar to those of fast-conducting fibers^22,41–44^. The absence of CaT alteration in the MSC co-cultures reinforces the hypothesis that cardiac fibroblasts specifically influence cardiomyocytes to specialize into fast-conducting fibers. Other studies have reported similar functional changes related to excitation–contraction coupling when cardiomyocytes are under the influence of cardiac fibroblasts^32,45–48^. Recently, Nagaraju et al. showed that myofibroblasts coupled with cardiomyocytes can also modulate cardiomyocyte action potentials^48^. Similarly, they observed hyperpolarization of the resting membrane potential; however, action potential duration was faster in cardiomyocytes coupled with myofibroblasts. These changes were mainly attributed to the arrhythmogenic influence of cardiac fibroblasts due to the down-regulation of ion channel expression and heterocellular coupling. Here we directly correlated the changes in CaTs with increased cardiomyocyte protein expression of VCS markers, suggesting specialization of cardiomyocytes into VCS-like cells.

In agreement with a VCS-like phenotype, our patch-clamp experiments revealed prolonged action potential duration in fCM compared to control cells, a hallmark of purkinje action potential in murine heart^49,50^. Furthemore, additional feature of purkinje cells is the presence of larger sodium currents when compared to cardiomyocytes, which is also supported by our molecular experiments^49,50^. Higher levels of sodium current leads to enhancement in the maximal dV/dt in the upstroke phase of action potential from purkinje cells^51^. Curiously, in our electrophysiological studies we observed decreased dV/dt in fCM compared to control group. It is known that sodium channel is found in plasma membrane of cells as a macromolecular complex and accessory subunits, such as β1–4 have major impact in the expression and function of the channel^52^. Thus additional signallling molecules may be necessary to give a complete functional sodium channel in fCM.

We have shown that cardiac fibroblasts can induce the VCS gene network in cardiomyocytes and that the fibroblast-conditioned media is sufficient to recapitulate this response. Co-cultures of cardiomyocytes and cardiac fibroblasts showed higher gene expression of the cardiogenic and VCS-relevant transcription factors *Irx3* and *Nkx2.5* when compared to cardiomyocyte cultures. Consequently, the expression of VCS-related downstream genes was also increased in the same co-cultures (i.e. *Scn5a*, connexins 40 and 45). Cardiac fibroblast-conditioned media additionally reduced in more than ten percent the proportion of cells presenting characteristics of ventricular working cardiomyocytes in the cardiomyocyte-enriched population (reduction in cardiomyocyte area and number of connexin- 43 positive cardiomyocytes).

Using cardiomyocyte organoids, we showed that cardiomyocytes on the outer surface of organoids receiving fibroblast-conditioned media displayed increased expression of connexin 40 and NAV1.5 compared to the control. These data emphasize the 3-dimensional patterning of the expression of VCS markers in cardiomyocytes under the influence of fibroblast-secreted factors that can gain access to inner layers only by diffusion. Notably, the fact that mainly the outer layer cardiomyocyte organoids had increased expression of VCS markers upon fCM treatment, suggests that the VCS-inducing fCM factors may have a local action, as usually observed for components of the extracellular matrix. Even though the existence of any influence mediated by cardiac fibroblast transmembrane proteins cannot be excluded, these results demonstrate that cardiac fibroblast-conditioned media is sufficient to induce the expression of VCS markers in cardiomyocytes in a paracrine fashion and exclude the possibility of cardiac fibroblasts being responsible for the increased expression of VCS-related genes in the mixed co-cultures rather than cardiomyocytes (Fig. 4).

We demonstrated that induction of cardiomyocytes into conduction cells in response to fibroblast-secreted factors was dependent on the activation of the NOTCH1 pathway in cardiomyocytes and fibroblasts, and independent of cell proliferation (Figure S5). Similarly, Rentschler et al. observed that newborn mice with NOTCH activated hearts have ectopic expansion of conductive tissue independent of cell proliferation. Furthermore, the conductive phenotype in murine newborn cardiomyocytes with activated NOTCH1 pathway was linked to changes in gene expression and prolongation of action potentials^24^. Thus, our data reinforce the known role of the NOTCH1 pathway for the induction of VCS-like cells and demonstrate the importance of cardiac fibroblasts for the activation of this pathway in cardiomyocytes. Interestingly, the secretion of these factors by cardiac fibroblasts was dependent on NOTCH1 activation in the fibroblasts themselves. Several studies also demonstrate the importance of the NOTCH pathway in fibroblasts and we also verified the expression of activated NOTCH1 in our fibroblast cultures (Data not shown)^53,54^. Previous analysis of cardiac fibroblast secretome before and after TGF-β treatment revealed that the main proteins in the cardiac fibroblast secretome are indeed involved in the extracellular matrix^27^. No canonical NOTCH ligands were identified in the secretomes of cardiac fibroblasts or TGF-β-induced myofibroblasts, suggesting that our observed NOTCH1 activation in cardiomyocytes upon fCM treatment is triggered by components of the extracellular matrix.

## Conclusion

**Figure 8:**
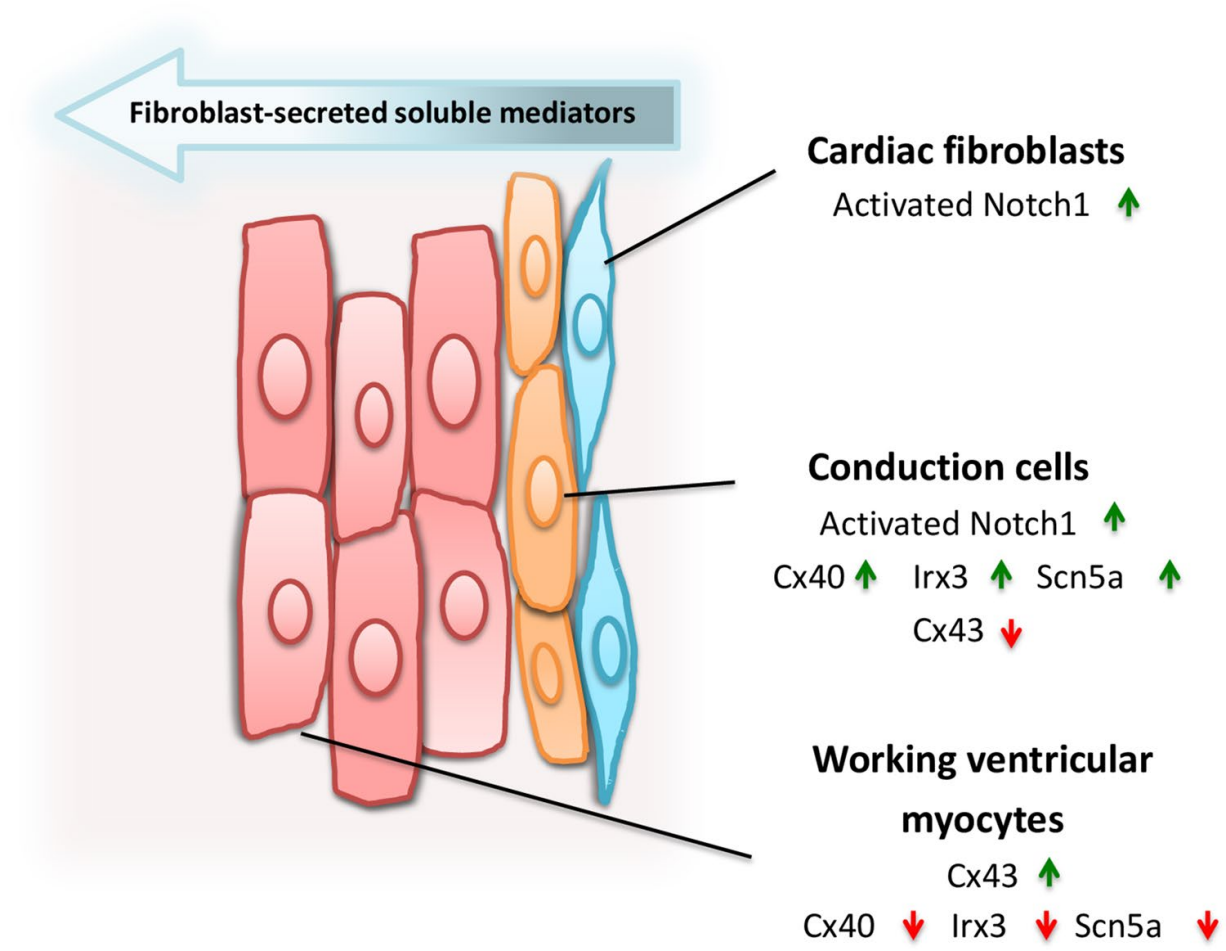
Cardiac fibroblast-mediated patterning of VCS-like cells in vitro. We have shown that cardiac fibroblasts from neonate rats induce molecular and functional changes characteristic of fast-conducting fibers in adjacent cardiomyocytes (E.g. increased expression of *Cx40*, *Irx3*, *Nkx2.5, Scn5a* and CONTACTIN 2, and longer calcium transients) and reduce characteristics of working cardiomyocytes (E.g. decreased cell area, Cx43 expression, and faster calcium transients). The cardiac fibroblast-conditioned media was sufficient to activate the NOTCH1 pathway and induce the expression of ventricular conduction system markers in cardiomyocytes. Interestingly, the secretion of these factors was dependent on the activation of the NOTCH pathway in fibroblasts; which sheds light on the central role that the NOTCH1 pathway plays in the intercommunication between fibroblasts and cardiomyocytes and the consequent induction of VCS-like phenotype in cardiomyocytes in a juxtacrine manner in vitro.

Proper embryonic development and postnatal maturation of the ventricular conduction system are key steps for the correct conduction of electrical impulses throughout the heart. The reported results provide evidence that cardiac fibroblast-secreted factors activate the NOTCH1 signaling to induce postnatal maturation of cardiomyocytes into VCS-like cells in vitro. The secretion of these factors by cardiac fibroblasts was also dependent on activation of the NOTCH1, highlighting the central role of the NOTCH1 in this crosstalk between cardiac fibroblasts and cardiomyocytes to induce VCS-like cells. Thus, we propose a model in which the NOTCH1 exerts a critical role in the crosstalk between cardiac fibroblasts and cardiomyocytes to give rise to VCS-like cells (Fig. 8). Future studies are needed to investigate whether this cellular interaction between cardiac fibroblasts and cardiomyocytes also takes place in vivo, to determine the secreted factors that cause this response, and if cardio myofibroblasts will result in qualitative or quantitative changes in this response under pathological conditions contributing to increased arrhythmogenic episodes and morbi-mortality associated to these events. Altogether, these findings highlight the possible role of cardiac fibroblasts in the development of VCS and may give new insights regarding the generation and targeting of arrhythmias associated to cardiac port-injury conditions.

## Supplementary information

Supplementary file1

Supplementary file2

Supplementary file3

Supplementary file4

Supplementary file5

## References

[1] Pinto AR (2016). Revisiting cardiac cellular composition. Circ. Res..

[2] Ieda M (2009). Cardiac fibroblasts regulate myocardial proliferation through β1 Integrin signaling. Dev. Cell.

[3] Hachiro T, Kawahara K, Sato R, Yamauchi Y, Matsuyama D (2007). Changes in the fluctuation of the contraction rhythm of spontaneously beating cardiac myocytes in cultures with and without cardiac fibroblasts. BioSystems.

[4] Furtado MB, Nim HT, Boyd SE, Rosenthal NA (2016). View from the heart: cardiac fibroblasts in development, scarring and regeneration. Development.

[5] Virágh S, Challice CE (1982). The development of the conduction system in the mouse embryo heart. Dev. Biol..

[6] Christoffels VM, Moorman AFM (2009). Development of the cardiac conduction system why are some regions of the heart more arrhythmogenic than others?. Circ. Arrhythmia Electrophysiol..

[7] Mohan RA, Boukens BJ, Christoffels VM (2018). Developmental origin of the cardiac conduction system: Insight from lineage tracing. Pediatr. Cardiol..

[8] van Weerd JH, Christoffels VM (2016). The formation and function of the cardiac conduction system. Dev..

[9] David S, Park GIF (2017). Development and function of the cardiac conduction system in health and disease. J. Cardiovasc. Dev. Dis..

[10] Kim KH (2016). Irx3 is required for postnatal maturation of the mouse ventricular conduction system. Sci. Rep..

[11] Miquerol L (2010). Biphasic development of the mammalian ventricular conduction system. Circ. Res..

[12] Meysen S (2007). Nkx2.5 cell-autonomous gene function is required for the postnatal formation of the peripheral ventricular conduction system. Dev. Biol..

[13] Cerrone M (2007). Arrhythmogenic mechanisms in a mouse model of catecholaminergic polymorphic ventricular tachycardia. Circ. Res..

[14] Caref E, Ben Boutjdir M, Himel HD, El-Sherif N (2008). Role of subendocardial Purkinje network in triggering torsade de pointes arrhythmia in experimental long QT syndrome. Europace.

[15] Kang G (2010). Purkinje cells from RyR2 mutant mice are highly arrhythmogenic but responsive to targeted therapy. Circ. Res..

[16] Vincentz JW (2020). Variation in a left ventricle-specific hand1 enhancer impairs GATA transcription factor binding and disrupts conduction system development and function. Circ. Res..

[17] van Eif VWW, Devalla HD, Boink GJJ, Christoffels VM (2018). Transcriptional regulation of the cardiac conduction system. Nat. Rev. Cardiol..

[18] Zhang SS (2011). Iroquois homeobox gene 3 establishes fast conduction in the cardiac His-Purkinje network. Proc. Natl. Acad. Sci. USA..

[19] Durocher D, Charron F, Warren R, Schwartz RJ, Nemer M (1997). The cardiac transcription factors nkx2-5 and GATA-4 are mutual cofactors. EMBO J..

[20] Linhares VLF (2004). Transcriptional regulation of the murine Connexin40 promoter by cardiac factors Nkx2-5, GATA4 and Tbx5. Cardiovasc. Res..

[21] Oyamada M, Takebe K, Oyamada Y (2013). Regulation of connexin expression by transcription factors and epigenetic mechanisms. Biochim. Biophys. Acta Biomembr..

[22] Harris BS (2006). Differentiation of cardiac Purkinje fibers requires precise spatiotemporal regulation of Nkx2-5 expression. Dev. Dyn..

[23] IPG M (2007). A molecular pathway including Id2, Tbx5, and Nkx2–5 required for cardiac conduction system development. Cell.

[24] Rentschler S (2012). Myocardial notch signaling reprograms cardiomyocytes to a conduction-like phenotype. Circulation.

[25] Milan DJ, Giokas AC, Serluca FC, Peterson RT, MacRae CA (2006). Notch1b and neuregulin are required for specification of central cardiac conduction tissue. Development.

[26] LaFoya B (2016). Notch: A multi-functional integrating system of microenvironmental signals. Dev. Biol..

[27] Abonnenc M (2013). Extracellular matrix secretion by cardiac fibroblasts: Role of MicroRNA-29b and MicroRNA-30c. Circ. Res..

[28] Howard CM, Baudino TA (2014). Dynamic cell–cell and cell-ECM interactions in the heart. J. Mol. Cell. Cardiol..

[29] Jensen L (2018). Integrated molecular, biochemical, and physiological assessment unravels key extraction method mediated influences on rat neonatal cardiomyocytes. J. Cell. Physiol..

[30] Zogbi C (2020). Beneficial effects of IL - 4 and IL - 6 on rat neonatal target cardiac cells. Sci. Rep.

[31] Fathi E, Farahzadi R (2016). Isolation, culturing, characterization and aging of adipose tissue-derived mesenchymal stem cells: A brief overview. Braz. Arch. Biol. Technol..

[32] Pedrotty DM, Klinger RY, Kirkton RD, Bursac N (2009). Cardiac fibroblast paracrine factors alter impulse conduction and ion channel expression of neonatal rat cardiomyocytes. Cardiovasc. Res..

[33] Dariolli R (2013). Porcine adipose tissue-derived mesenchymal stem cells retain their proliferative characteristics, senescence, karyotype and plasticity after long-term cryopreservation. PLoS ONE.

[34] Zogbi C (2014). Early postnatal rat ventricle resection leads to long-term preserved cardiac function despite tissue hypoperfusion. Physiol. Rep..

[35] Carneiro AP, Fonseca-Alaniz MH, Dallan LAO, Miyakawa AA, Krieger JE (2017). β-arrestin is critical for early shear stress-induced Akt/eNOS activation in human vascular endothelial cells. Biochem. Biophys. Res. Commun..

[36] Remme CA (2009). The cardiac sodium channel displays differential distribution in the conduction system and transmural heterogeneity in the murine ventricular myocardium. Basic Res. Cardiol..

[37] Pallante BA (2010). Contactin-2 expression in the cardiac Purkinje fiber network. Circ. Arrhythmia Electrophysiol..

[38] Chatelier A (2012). A distinct de novo expression of Nav15 sodium channels in human atrial fibroblasts differentiated into myofibroblasts. J. Physiol..

[39] Terada R (2011). Ablation of Nkx2-5 at mid-embryonic stage results in premature lethality and cardiac malformation. Cardiovasc. Res..

[40] Ono N (2009). Morphological varieties of the purkinje fiber network in mammalian hearts, as revealed by light and electron microscopy. Arch. Histol. Cytol..

[41] Tsai SY (2015). Efficient generation of cardiac purkinje cells from ESCs by activating cAMP signaling. Stem Cell Rep..

[42] Gintant GA, Datyner NB, Cohen IS (1984). Slow inactivation of a tetrodotoxin-sensitive current in canine cardiac Purkinje fibers. Biophys. J..

[43] Robinson RB, Boyden PA, Hoffman BF, Hewett KW (1987). Electrical restitution process in dispersed canine cardiac Purkinje and ventricular cells. Am. J. Physiol. Hear. Circ. Physiol..

[44] Persson F, Andersson B, Duker G, Jacobson I, Carlsson L (2007). Functional effects of the late sodium current inhibition by AZD7009 and lidocaine in rabbit isolated atrial and ventricular tissue and Purkinje fibre. Eur. J. Pharmacol..

[45] Mayourian J (2018). Physiologic, pathologic, and therapeutic paracrine modulation of cardiac excitation-contraction coupling. Circ. Res..

[46] Sridhar S, Vandersickel N, Panfilov AV (2017). Effect of myocyte-fibroblast coupling on the onset of pathological dynamics in a model of ventricular tissue. Sci. Rep..

[47] Sullivan KE, Black LD (2013). The role of cardiac fibroblasts in extracellular matrix-mediated signaling during normal and pathological cardiac development. J. Biomech. Eng..

[48] Nagaraju CK (2019). Myofibroblast modulation of cardiac myocyte structure and function. Sci. Rep..

[49] Vaidyanathan R (2013). The ionic bases of the action potential in isolated mouse cardiac Purkinje cell. Hear. Rhythm.

[50] Watanabe T, Delbridge LM, Bustamante JO, McDonald TF (1983). Heterogeneity of the action potential in isolated rat ventricular myocytes and tissue. Circ. Res..

[51] Berecki G, Wilders R, de Jonge B, van GinnekenGinneken ACG, Verkerk AO (2010). Re-evaluation of the action potential upstroke velocity as a measure of the na+ current in cardiac myocytes at physiological conditions. PLoS ONE.

[52] O’Malley HA, Isom LL (2015). Sodium channel β subunits: Emerging targets in channelopathies. Annu. Rev. Physiol..

[53] Sassoli C (2013). Relaxin prevents cardiac fibroblast-myofibroblast transition via notch-1-mediated inhibition of TGF-β/Smad3 signaling. PLoS ONE.

[54] Fan YH (2011). Notch signaling may negatively regulate neonatal rat cardiac fibroblast-myofibroblast transformation. Physiol. Res..

