# Supplementary material for: NOTCH1 is critical for fibroblast-mediated induction of cardiomyocyte specialization into ventricular conduction system-like cells in vitro
Source: Sci Rep. 2020 Sep 30;10:16163. doi: 10.1038/s41598-020-73159-0 (PMC7527973; doi:10.1038/s41598-020-73159-0)
Supplement: Supplementary file 1 — Supplementary file1 [file 41598_2020_73159_MOESM1_ESM.docx]

**Supplementary information**

**NOTCH1 is critical for fibroblast-mediated induction of cardiomyocyte specialization into ventricular conduction system-like cells *in vitro***

**Agatha Ribeiro da Silva**^1^**, Elida A. Neri**^1^**, Lauro Thiago Turaça**^1^**, Rafael Dariolli**^1^**, Miriam H. Fonseca-Alaniz**^1^**, Artur Santos Miranda^2^, Danilo Roman Campos^2^_,_ Gabriela Venturini**^1^**, Jose E. Krieger**^1^

^1^Instituto do Coracao (InCor) da Faculdade de Medicina da Universidade de Sao Paulo (FMUSP)

^2^Paulista School of Medicine, Federal University of São Paulo (EPM-UNIFESP)

**Figure S1. Characterization of neonatal rat cardiac fibroblasts.** Immunofluorescence analysis of fibroblast and myofibroblast markers. All neonatal rat cardiac fibroblasts were used in passage 1 in order to inhibit differentiation into myofibroblasts. (A) Vimentin-labeled fibroblast culture (in green), fibroblast marker. Fibronectin-labeled fibroblast culture (in red), fibroblast marker. CD90-labeled fibroblast culture (in magenta), fibroblast marker. PDGFRα-labeled fibroblast culture (in cyan), fibroblast marker. Smooth muscle alpha actin labeled fibroblast culture, αSMA (in cyan), marker for fibroblast differentiation into myofibroblasts. (B) Fibroblast (Fb) and myofibroblast (positive control) cultures were stained for collagen using the PicroSirius red staining kit (Chondrex). Scale = 20μm.

**Figure S2. Calcium transient analysis of cardiomyocyte-enriched areas.** Cardiomyocytes were plated in the center of our spatially delimited co-culture system and either cardiac fibroblasts or mesenchymal stem cells were plated on the outside area. The calcium transients of contractile cells in the cardiomyocyte-enriched areas were recorded and measured. (A) CaT mean curves for each experimental group. (B) Normalized CaT amplitudes. (C) Time to reach 50% of calcium decay (CaD50). (D) time to reach 90% of calcium decay (CaD90). (E) Time to reach the maximum peak of calcium per beating cycle (Time to Peak). (F) Time to reach basal levels of Calcium after maximum peak (Ca decay time). (G) The Resting interval or the time between the basal level reached after contraction until the new CaT. The cardiomyocyte-enriched area in the center of the cardiomyocyte-only coverslip was used as the control. *p < 0.05, ** p < 0.01, *** p < 0.001 after Bonferroni *post-hoc* test (alpha 0.05). Sample sizes were 15 cells of Cardiomyocytes (center), 12 cells of Cardio/Fibro (contact) and 10 cells of Cardio/rMSCs (contact).

**Figure S3. Demonstrative calcium transient curves.** Representative calcium transients of the cardiomyocytes in the center of delimited co-cultures, and cardiomyocytes in the contact zone with either cardiac fibroblasts or MSCs.

**Figure S4. Assessment of organoid viability seven days after plating.** Live cells are shown in green and dead cells are label in red. Organoids treated with methanol were used as positive controls for cell death. (A-C) Cardiomyocyte organoids treated with control media. (A’-C’) Cardiomyocyte organoids treated with fCM. Scale bars=100 μm.

**Figure S5. Induction of VSC-like cells by cardiac fibroblasts is independent of cell proliferation.** Proliferation analysis by high content screening 48h after plating. (A-H’) Demonstrative immunofluorescence images. Cell nuclei (DAPI), cardiomyocytes (cardiac Troponin I positive cells, Red), proliferative cells as ki67 positive (magenta), and cardiac fibroblasts as troponin I negative cells. Scale bars = 20µm. (B-C) Percentage of proliferating Ki67 positive cardiomyocytes and fibroblasts, respectively. Results are disposed as a mean ± SEM. Data were subjected to one-way ANOVA, followed by multiple comparisons against the control group; p<0.05 was considered significant. N=6 (each consisting of more than 900 cells).

**Figure S6. Extended blots.** (A) Figure 5H. (B) Figure 5I. (C) Figure 7B, (D) Figure 7C e (E) Figure 7D.

**Table 1. List of primers for real time RT-PCR.** RT-PCR for the *Irx3* and *Scn5a* genes were performed using TaqMan probes.

**Figure S1
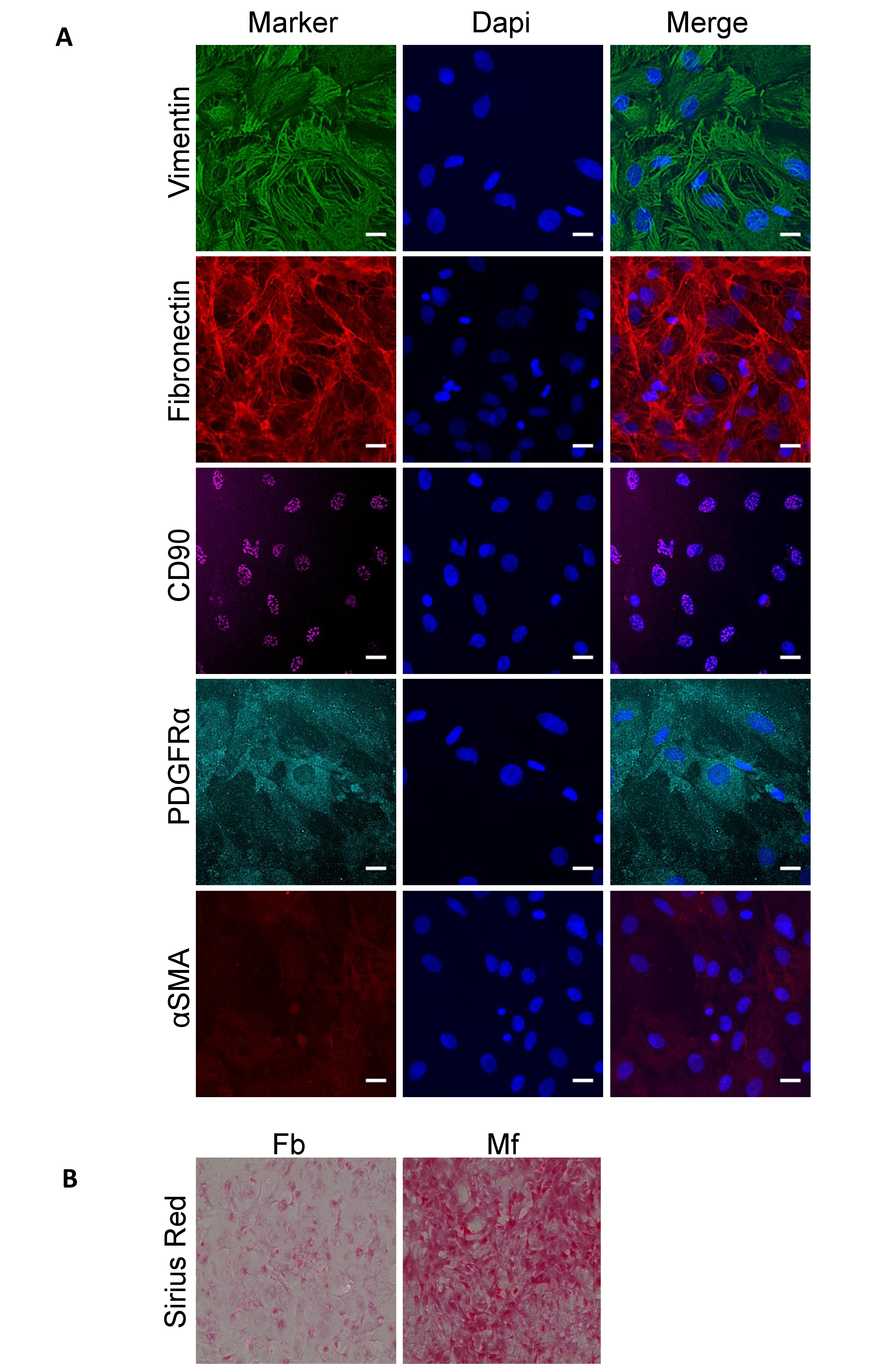
**

**Figure S2**
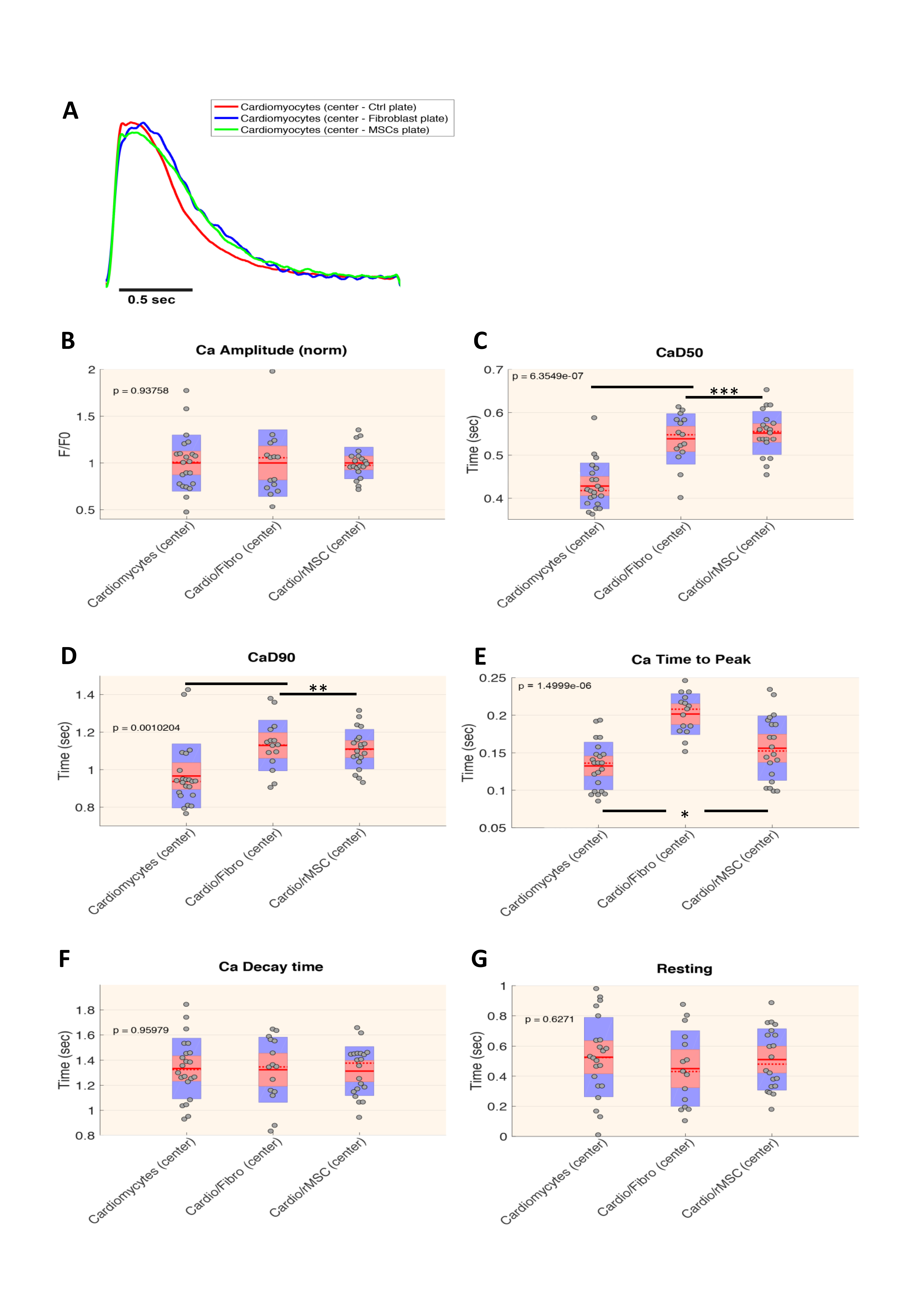


**Figure S3**


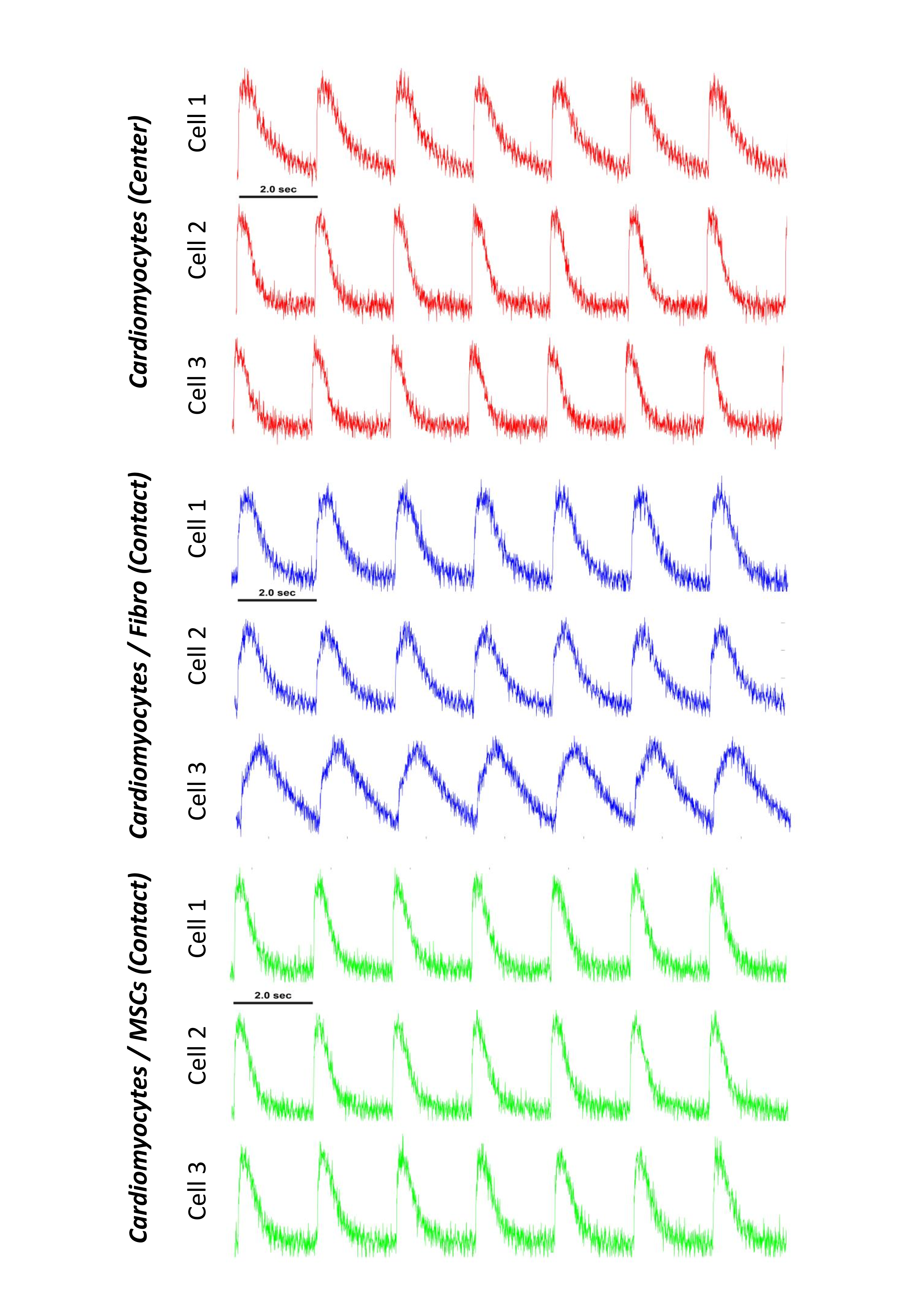


**Figure S4**
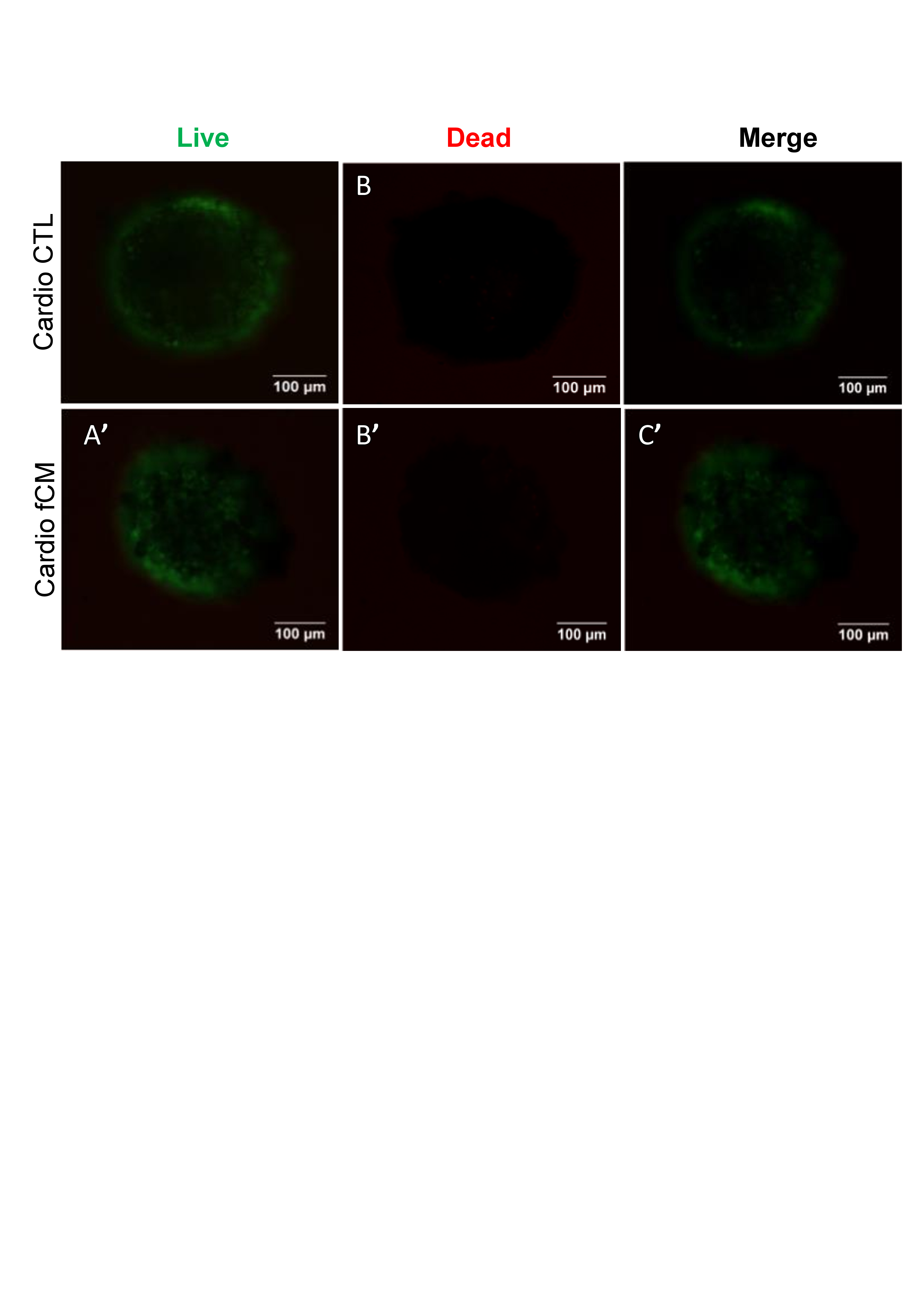


**Figure S5**


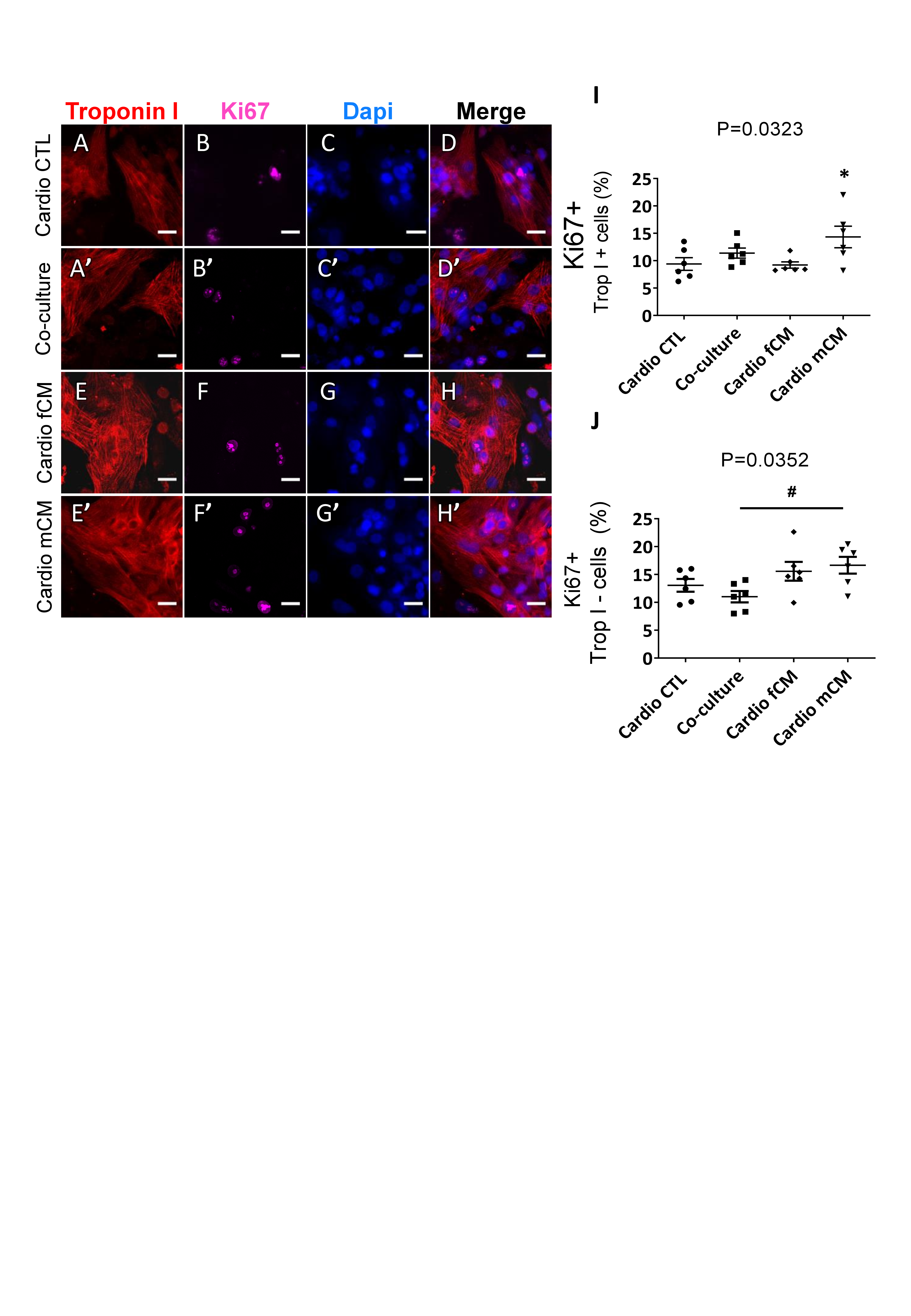


**
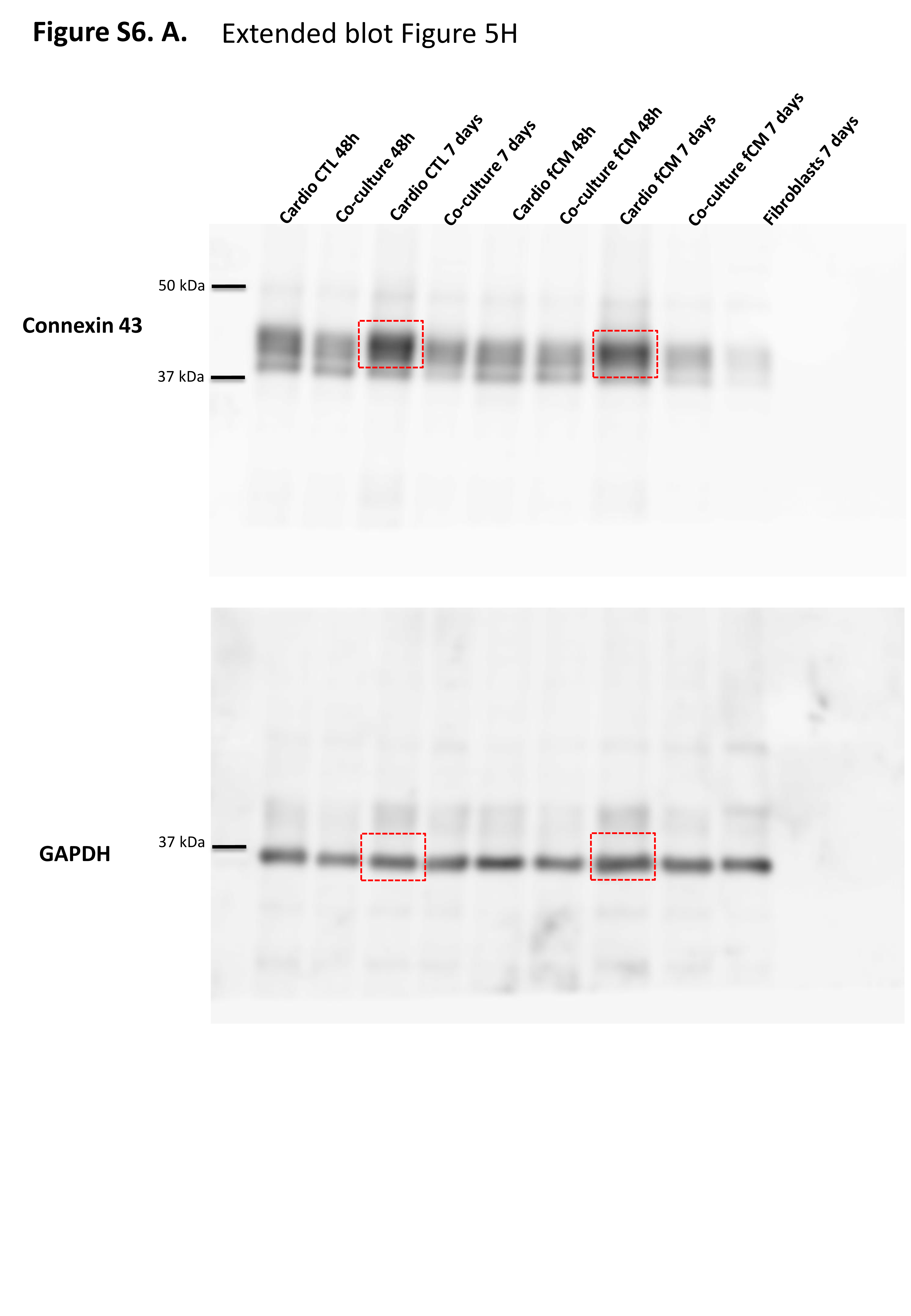
**

**
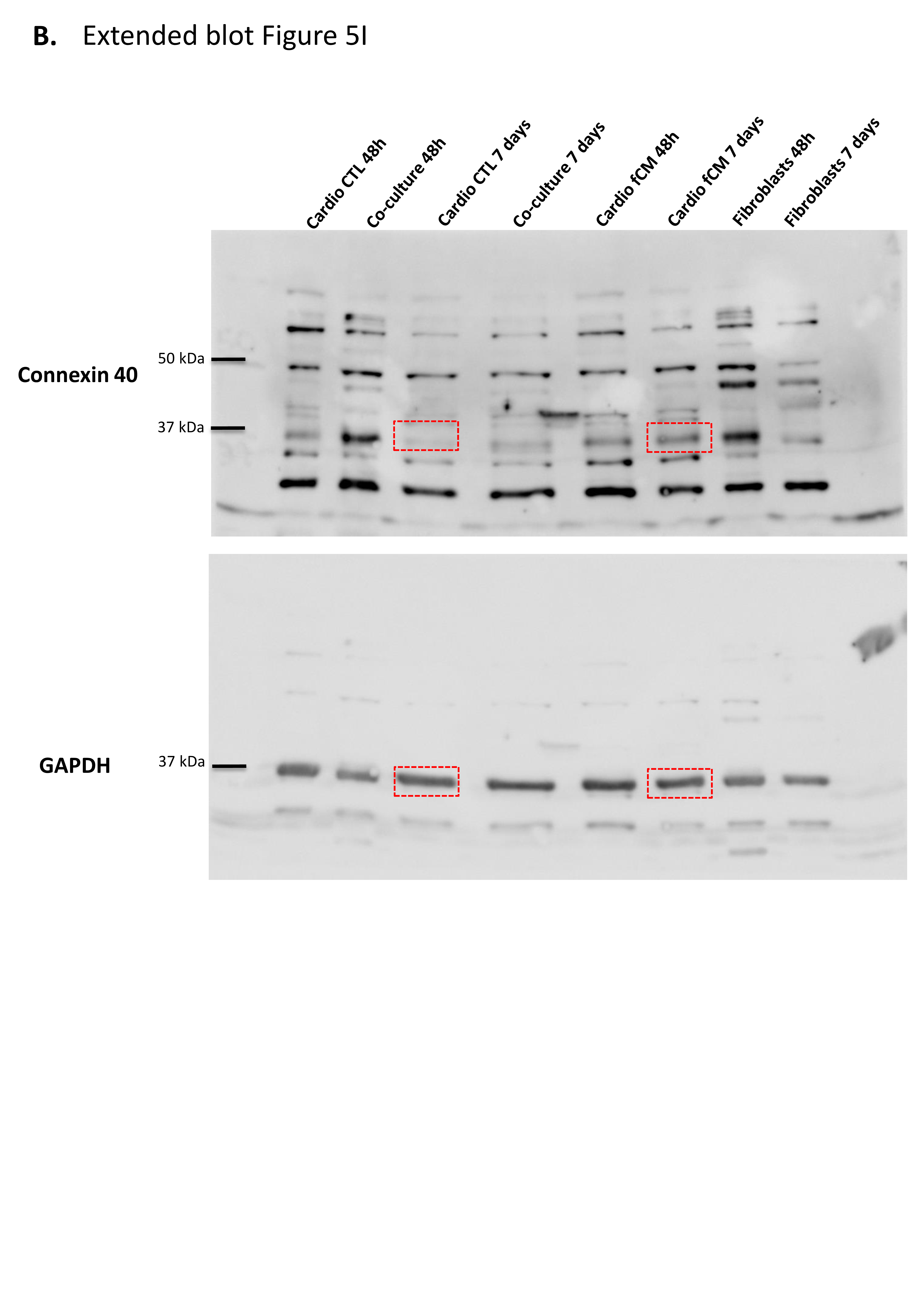
**

**
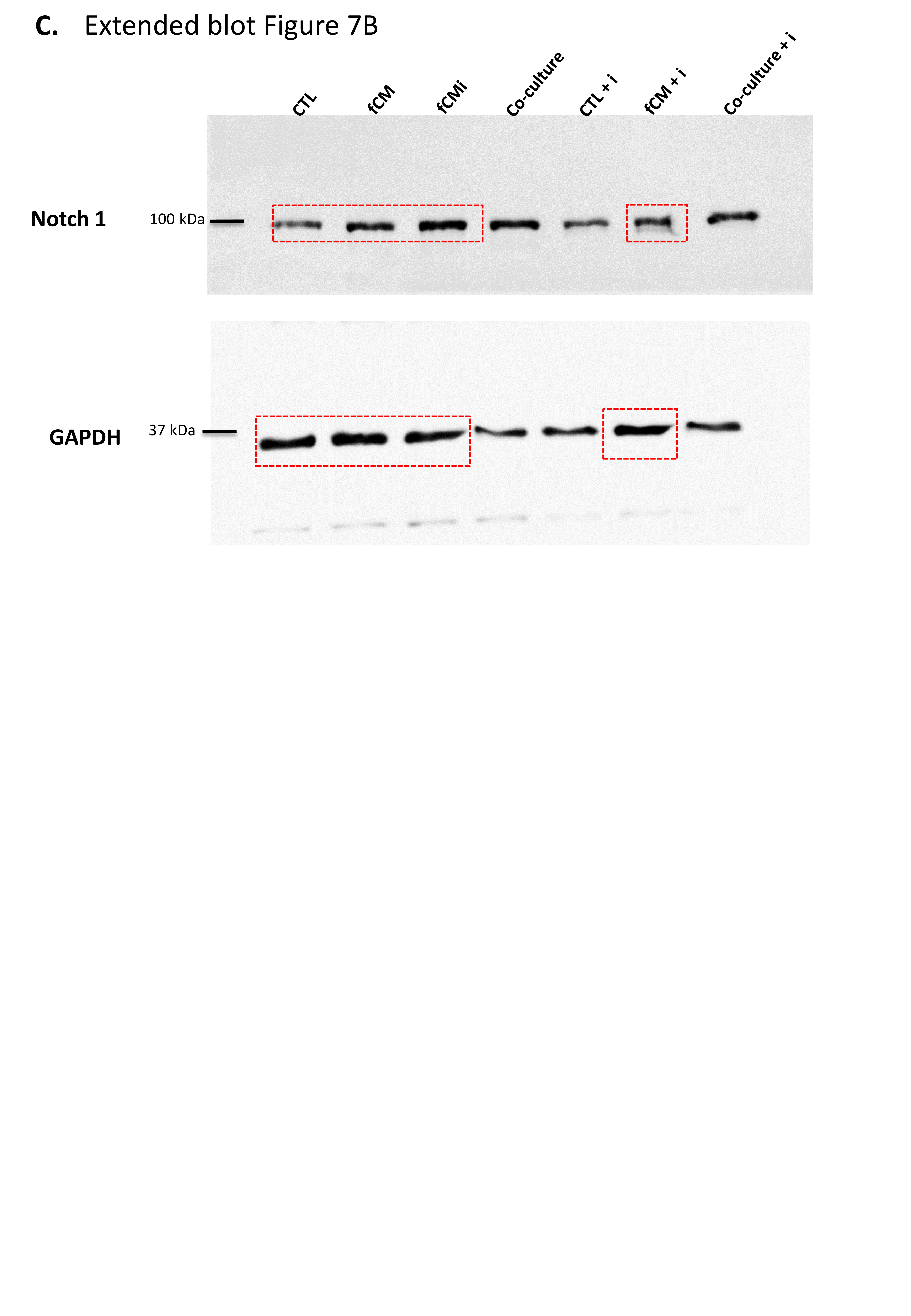
**

**
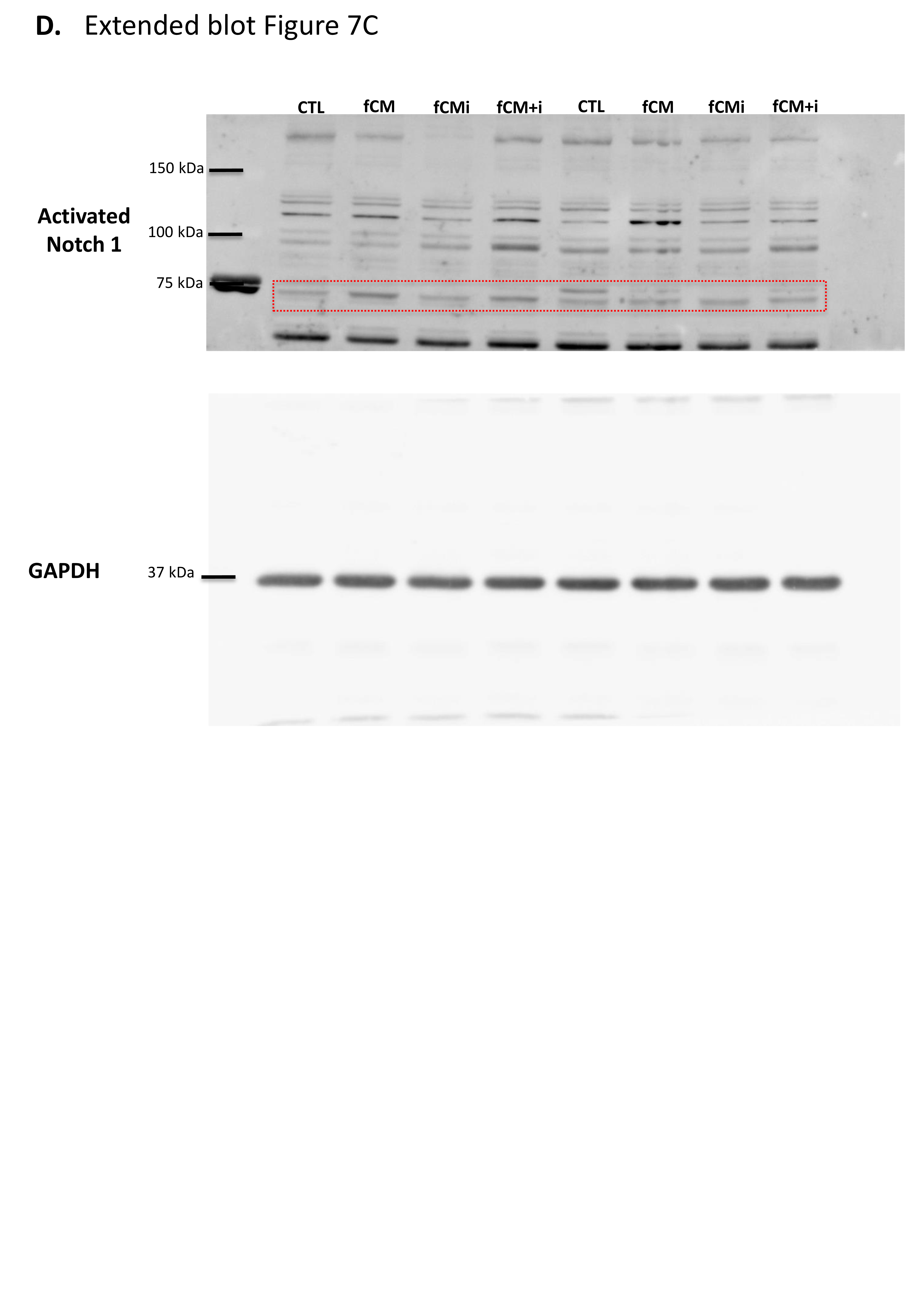
**

**
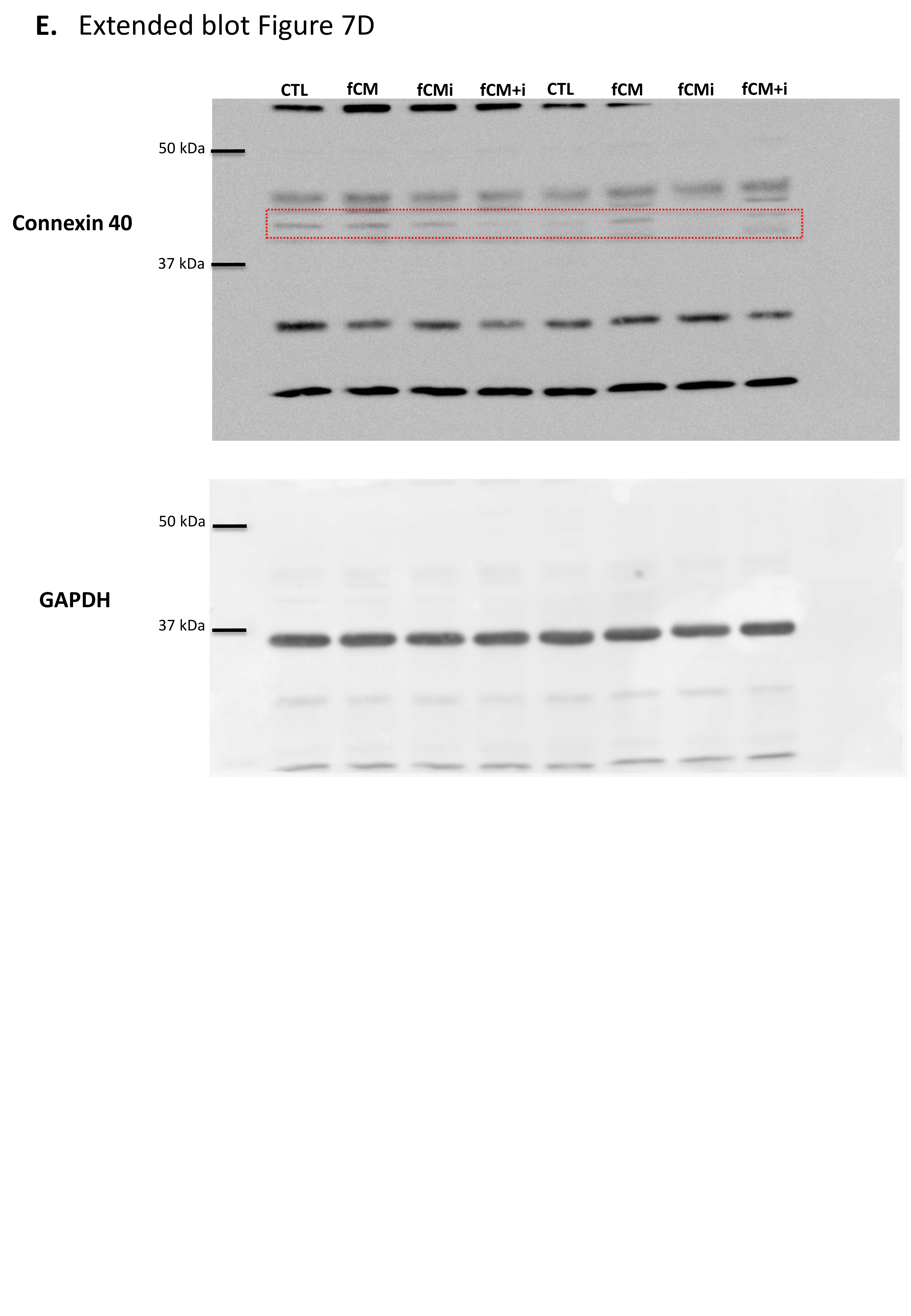
**

**

**
