# Supplementary figures and images for: NOTCH1 is critical for fibroblast-mediated induction of cardiomyocyte specialization into ventricular conduction system-like cells in vitro
Source: Sci Rep. 2020 Sep 30;10:16163. doi: 10.1038/s41598-020-73159-0 (PMC7527973; doi:10.1038/s41598-020-73159-0)

## Slide 1
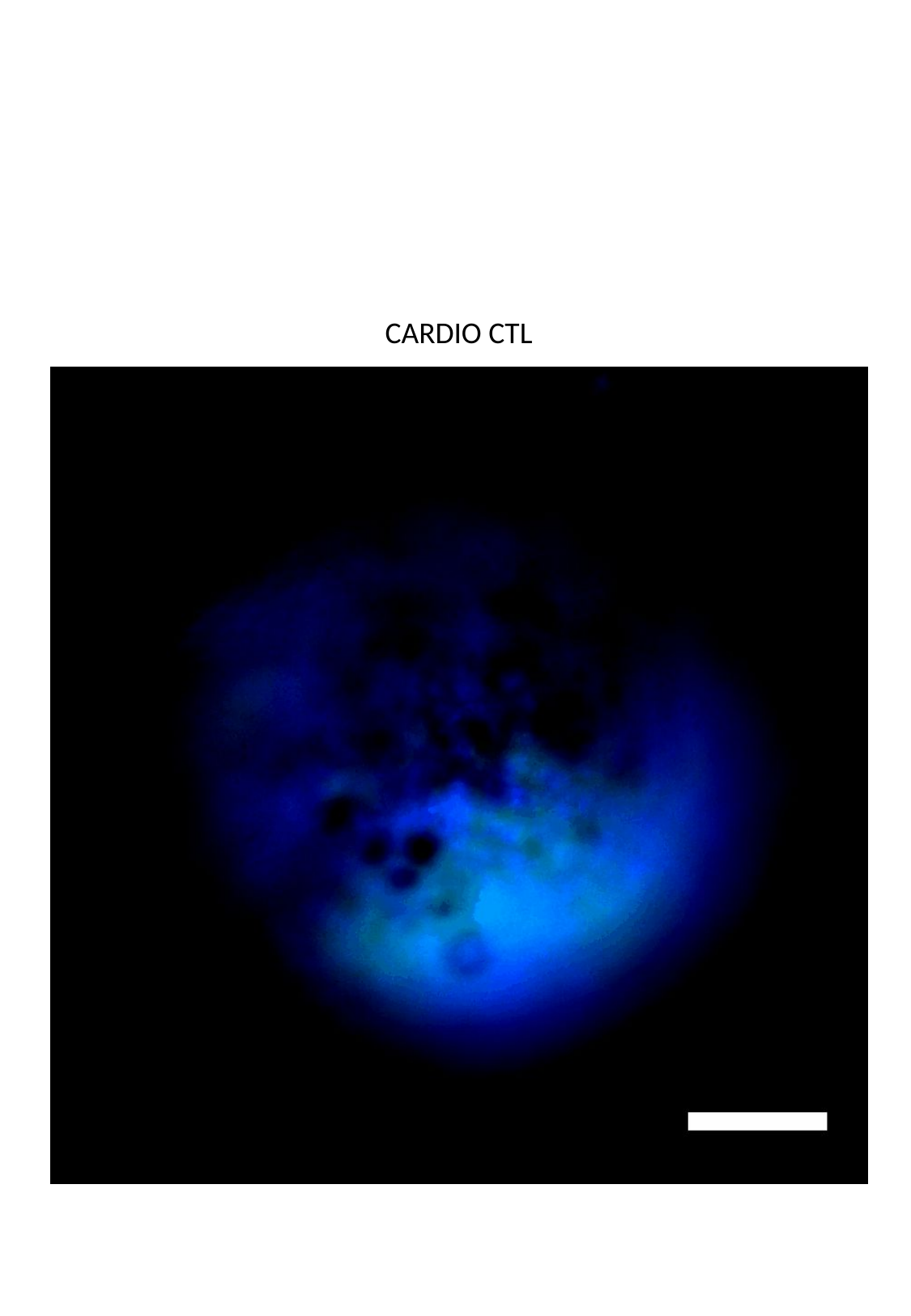

CARDIO CTL

Supplement: Supplementary file 2 — Supplementary file2 [file 41598_2020_73159_MOESM2_ESM.pptx]
